# Automatic Clustering of Excited-State Trajectories: Application to Photoexcited Dynamics

**DOI:** 10.1021/acs.jctc.3c00776

**Published:** 2023-09-13

**Authors:** Kyle Acheson, Adam Kirrander

**Affiliations:** † EaStCHEM, School of Chemistry and Centre for Science at Extreme Conditions, University of Edinburgh, David Brewster Road, Edinburgh EH9 3FJ, U.K.; ‡ Physical and Theoretical Chemistry Laboratory, Department of Chemistry, University of Oxford, South Parks Road, Oxford OX1 3QZ, U.K.; § Department of Chemistry, University of Warwick, Coventry CV4 7AL, U.K.

## Abstract

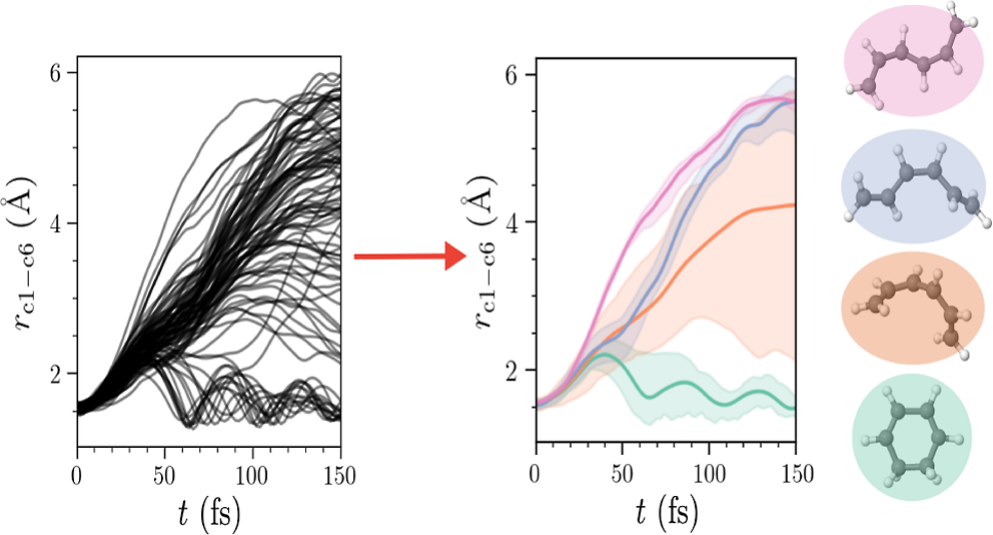

We introduce automatic clustering as a computationally efficient tool for classifying and interpreting trajectories from simulations of photo-excited dynamics. Trajectories are treated as time-series data, with the features for clustering selected by variance mapping of normalized data. The L_2_-norm and dynamic time warping are proposed as suitable similarity measures for calculating the distance matrices, and these are clustered using the unsupervised density-based DBSCAN algorithm. The silhouette coefficient and the number of trajectories classified as noise are used as quality measures for the clustering. The ability of clustering to provide rapid overview of large and complex trajectory data sets, and its utility for extracting chemical and physical insight, is demonstrated on trajectories corresponding to the photochemical ring-opening reaction of 1,3-cyclohexadiene, noting that the clustering can be used to generate reduced dimensionality representations in an unbiased manner.

## 1 Introduction

Photoexcited dynamics is complex, with the nuclear wave packet evolving across coupled multidimensional potential energy surfaces (PESs) on ultrashort timescales. Experimental techniques such as time-resolved spectroscopy, scattering, or Coulomb explosion imaging^1−5^ can offer invaluable insights, especially when combined with theory and simulations. Quite often, the key contributions to the experimental observable are attributed with the help of trajectory-based simulations using on-the-fly quantum dynamics or mixed quantum-classical methods such as surface hopping,^6^ ab initio multiple spawning,^7^ multiconfigurational Ehrenfest,^8,9^ and direct dynamics variational multiconfigurational Gaussian.^10^ The counterpoint to the increasing sophistication and accuracy of the simulations is the challenge of finding simple interpretations, which is not straightforward given that the trajectories encompass the inherent multidimensional complexity of the dynamics. In this context, an unbiased and objective method for arriving at an understanding of the key features of the dynamics, ideally also in terms of a reduced dimensionality model, would have great utility.

In recent years, computational chemistry has seen an increase in unsupervised and supervised machine learning algorithms.^11^ Moreover, unsupervised clustering algorithms that aim to identify key patterns and predict future observations have seen broad success in a wide range of fields. Applications to time-dependent data include the analysis of global positioning system data,^12^ the tracking of patterns in animal migration,^13^ the categorization of medical time-series data,^14^ and the deduction of trends in financial data and associated forecasting.^15^ In light of this, we explore the application of clustering algorithms capable of automatically categorizing the key aspects of trajectory data, providing a better overview of the multidimensional dynamics. This allows for the identification and classification of the decay or reaction pathways available to an excited system, with clusters of similar trajectories representing concentrations of reaction flux. Not only does this aid mechanistic understanding of simulations, but it also helps the interpretation of experimental observations and may assist in the inverse problem of constructing a molecular model commensurate with time-resolved experimental data.^16−21^

The complexity and computational cost of calculating accurate observables from simulations explains the persistence of simple approximations such as the independent atom model^22,23^ or Dyson norm calculations^24^ for scattering and photoelectron spectroscopy, respectively. Highly accurate calculations of total scattering signals from ab initio wavefunctions^25−28^ or photoelectron spectra using sophisticated R-matrix codes^29^ are computationally expensive. Clustering allows a reduced dimensionality model of the dynamics to be constructed using representative trajectories from each cluster. This model, in turn, can be used to calculate observables.^30^ Thus, we anticipate that effective clustering will enable more accurate calculations of observables.

While clustering algorithms have been used extensively to cluster molecular geometries taken from (ground electronic state) classical molecular dynamics simulations and have seen success in classifying the microstates sampled by the trajectory, e.g., in terms of protein conformations,^31−35^ the problem of clustering trajectories from excited-state simulations poses unique challenges. First of all, the task is not to cluster individual molecular geometries but rather continuous temporal sequences of geometries. The density and spatial distribution of the trajectories can vary significantly, and since the trajectories propagate across different electronic states and across different parts of the topography of each PES, their characteristic motions and frequencies can vary considerably. Furthermore, as copropagating trajectories transition between different electronic states, either by internal conversion or intersystem crossing, they can diverge, reflecting the branching of the nuclear wave packet. Finally, in contrast to standard force-field-based molecular dynamics simulations, the chemical structure of molecules during photoexcited processes often changes as bonds are made or broken. All these aspects make clustering in the context of photochemical reactions an interesting, challenging, and important problem.

The rest of this paper is structured as follows. In Section 2, we introduce trajectory data that models the electrocyclic ring-opening dynamics of 1,3-cyclohexadiene (CHD), followed by a discussion of the data preprocessing and clustering procedure. In Section 3, we discuss the results of applying clustering algorithms to the trajectory data and the limitations encountered. Finally, in Section 4, we present the conclusions and future avenues for further exploration.

## 2 Methods

Generally, a clustering algorithm aims to categorize a large dataset into distinct clusters, such that the intracluster variance is minimized and the intercluster variance is maximized.^36^ The application of clustering algorithms to time-dependent data to identify temporal patterns and characteristics is inherently more challenging than time-independent applications. This is a consequence of the fact that data points are no longer independent and that correlation between points in the same temporal sequence must be considered. In addition, different sequences may have different lengths, with local variations in timing or temporal alignment, and typically have high dimensionality.

### 2.1 Model Trajectories

**Figure 1:**
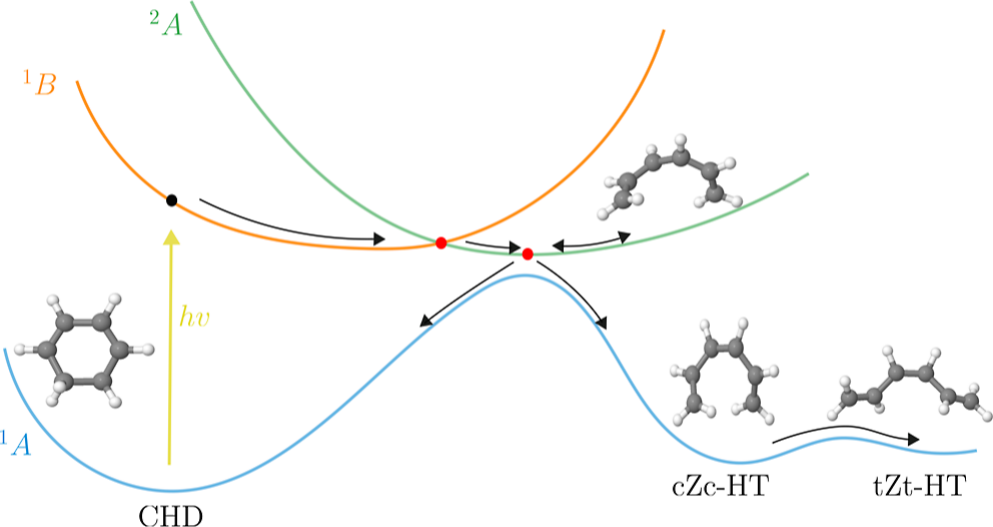
Photoinduced electrocyclic ring-opening of CHD to HT. The diabatic electronic states are shown schematically, with red points indicating conical intersections. Following formation of cZc-HT on the ground state, several cis/trans HT isomers are accessible.

**Figure 2:**
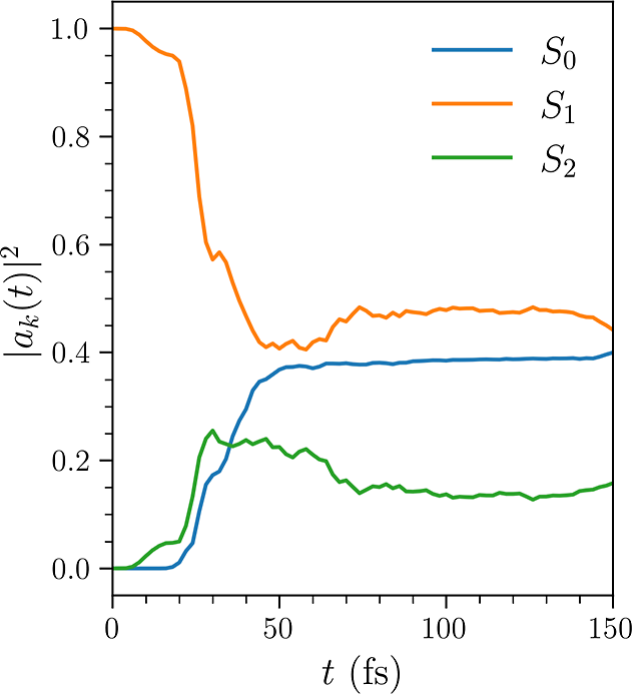
Net populations  calculated as an average over the full set of *N*_trj_ = 100 AIMCE trajectories for each of the *k* = 0–2 electronic states as a function of time. At early times, the S_1_ state corresponds to the diabatic 1B state. At 30 fs, a maxima in population of the S_2_ state is observed as the wave packet passes through the 2A/1B intersection, at subsequent times, the S_1_ state is of 2A character.

To test the application of clustering algorithms to photoexcited trajectory data, we utilize an ensemble of 100 semi-classical ab initio multiconfigurational Ehrenfest (AIMCE) trajectories that model the ring-opening reaction of CHD to 1,3,5-hexatriene (HT), with the electronic structure calculated at the SA3-CASSCF(6,4)/cc-pVDZ level, a brief overview of the AIMCE method is given in the Supporting Information. These trajectories are taken from ref (37). The overall physical situation is illustrated in Figure 1. Each trajectory evolves in time across the 36 internal geometry coordinates and the populations of the three electronic states considered in the dynamics. Figure 2 shows the populations of the three adiabatic singlet states included over the 150 fs simulation period.

The ring-opening of CHD to HT is a prototypical Woodward–Hoffmann electrocyclic reaction,^38−40^ which has been the subject of extensive theoretical and experimental studies.^30,37,41−64^ Following excitation to the S_1_ (1B) electronic state, the wave packet rapidly accelerates out of the Franck–Condon region, with the conrotatory twist of the terminal carbon atoms already taking place before the 2A/1B conical intersection (CoIn) is reached.^46,49^ The lifetime of the 1B state is on the order of 30 fs,^55,59^ as indicated by the clear maxima in S_2_ population in Figure 2. At the 2A/1B CoIn, the wave packet bypasses a cusp between the surfaces, forcing it to break symmetry via a set of symmetry equivalent 2A/1A CoIns.^43,46−48^ The acceleration from the slope of the cusp region results in rapid decay to the ground S_0_ state, forming either HT (initially cZc-HT) or returning to vibrationally hot CHD.

In Figure 2, we observe the onset in S_0_ population rise at 25 fs. The S_0_ population continues to rise as the S_1_ and S_2_ states stabilize at around 70 fs, by which time a large portion of the wave packet has reached the S_0_ ground state. Since the formation of cZc-HT occurs with an energy in excess of the barrier to isomerization on the ground state, the full range of cZc-, cZt-, and tZt-HT isomers appear. At times greater than 70 fs, the AIMCE simulations as shown in Figure 2 show that the populations stabilize with significant S_1_ population. At these intermediate times, the S_1_ state is of 2A character, and the remainder of the wave packet undergoes several more oscillations along the near flat 2A surface, causing it to disperse. This results in a broader distribution of geometries that have a higher C_1_–C_6_ torsion angle, which eventually decay to the ground state, potentially resulting in a small fraction of the more open E-HT isomers.^50^ The majority of experimental and theoretical studies estimate the total excited-state lifetime to be on the order of 130–140 fs.^46,51,55,58^ Generally, the quantum yield of HT has been reported to lie in the range of 40–60%.^44,51,54,59^

### 2.2 Feature Selection

In high-dimensional problems, such as the trajectories reported here, one must identify a small sample of data features on which clustering can be performed. This is largely a result of the curse of dimensionality, with the volume spanned by the data increasing exponentially with the number of features considered. Furthermore, inclusion of a number of similar and highly correlated features may bias the clustering result and lead to problems with interdependencies. If features that are irrelevant to the key trends in the data are included, overfitting may also occur, preventing the algorithm from identifying the underlying structure of the data.

The selection of a suitable feature set for time-dependent data presents additional challenges due to temporal correlations between features. Fortunately, a wide range of methods for feature selection exist based on chi-squared tests, variance thresholding, covariance analysis, regularization, or decision trees.^36,65^ Alternatively, dimensionality reduction techniques can determine the representation of the data that encodes the highest variance features, for instance, using principal component analysis (PCA),^66^ embedding methods, and autoencoders (AE).^67−69^ Note that while PCA is commonly employed in clustering analysis, the selected principal components are often not suitable for describing the dynamic evolution of a system across all time steps.

**Figure 3:**
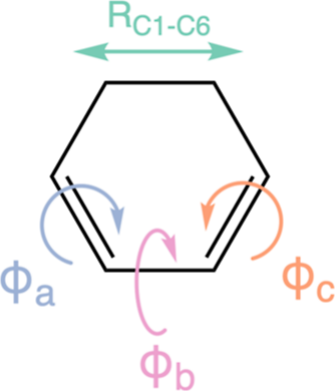
High variance internal coordinates that form the basis of the feature space on which clustering is performed. The selection includes the dihedral angles ϕ_a_, ϕ_b_, and ϕ_c_, as well as the bond length *R*_C1–C6_.

In this work, we employ a variance thresholding technique in which a minimal number of high variance features are selected for clustering. The benefit of this approach lies in its simplicity. While trajectory data that models photoexcited dynamics often includes a large number of coupled degrees of freedom, many of these may be redundant, and quite often key nuclear coordinates can be identified by the inspection of their variance over time. In the present case, plain internal coordinates are used as the basis, although we note that in more complicated cases, alternative representations, such as curvilinear coordinates, may provide better clustering results. In the case of CHD, we select a total of four high variance internal coordinates, as shown in Figure 3. Dihedral angles ϕ_a_ and ϕ_c_ are crucial in describing the isomerization of cZc-HT to tZt-HT on the ground state, whereas dihedral angle ϕ_b_ describes the out-of-plane torsion exhibited by the molecule along the near flat 2A electronic state at later times. As the three dihedral angles do not allow the distinction between cZc-HT from vibrationally hot CHD, we also include the C_1_–C_6_ bond.

### 2.3 Choice of Methodology

A number of approaches are available to tackle the problem of identifying the temporal trends in time-dependent data. The appropriate choice depends on the characteristics of the data in question. Typically, data, such as stock prices,^15^ temperature measurements,^70^ and some specific types of medical data (e.g. electrocardiograms^71^ and MRI observations^72^), are modeled as time series consisting of observations at regular time intervals.^73,74^ In contrast, trajectory data is usefully considered as a group of moving objects, such as vehicles,^12,75^ people,^76^ or animals,^13^ with each trajectory describing a particular evolution of associated spatial coordinates over time.^77,78^

**Figure 4:**
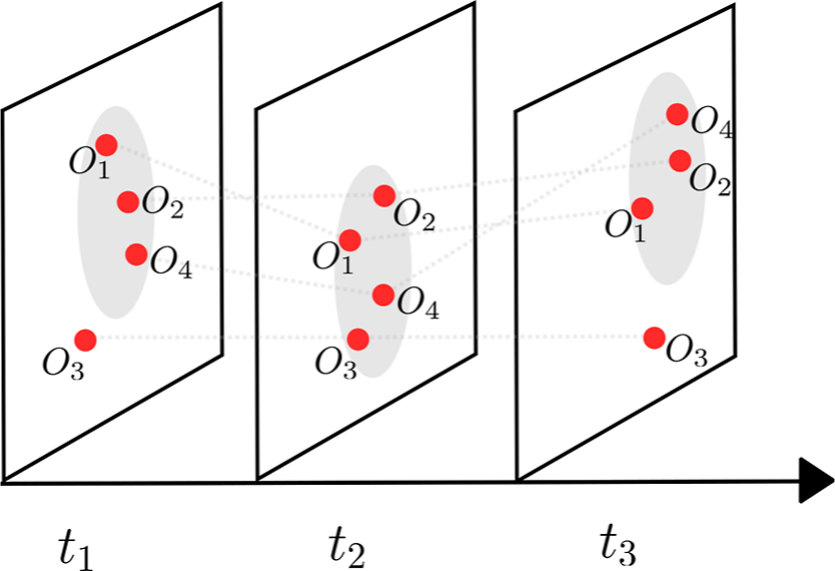
Schematic representation of the mining of spatiotemporal clusters. The evolution of four trajectories, [*o*_1_, *o*_2_, *o*_3_, *o*_4_], in a two-dimensional feature space is shown at three times, *t*_1_, *t*_2_, and *t*_3_, each represented by a slice. The gray ellipses mark the spatial clusters identified by application of a spatial clustering algorithm at each time independently.

Considering the trajectories as a group of moving objects, the spatiotemporal approach to clustering involves applying a clustering algorithm to each independent time step of the trajectories, generating a database of independent spatial clusters at each time.^77^ This is represented schematically in Figure 4, which shows a snapshot of four trajectories at three different times. At each time, the trajectories clustered together are contained within the gray ellipse. The temporal patterns are then mined from the resulting database of clusters by identifying the groups of trajectories that remain clustered across time. For example, in Figure 4, there is a clear spatiotemporal correlation between trajectories [*o*_1_, *o*_2_, *o*_4_] across all three times. More generally, there will exist a multitude of possible definitions of spatiotemporal clusters. Thus, the mining of temporal patterns will depend on the constraints provided for the chosen algorithm. Moving clusters, for instance, define a spatiotemporal cluster as a sequence of spatial clusters that meet a minimum overlap threshold over a specified number of consecutive time steps, whereas convoys are defined by the minimum number of trajectories continually clustered together over time. However, such approaches toward mining spatiotemporal patterns are often challenging and can be computationally demanding given that one must cluster each time step independently first.

An alternative approach is to consider the trajectory data as a multivariate time series, in which each coordinate is represented by its own sequence (time series).^73^ Although dependent on the chosen similarity measure and overall length of the time series, this approach is generally simpler and faster than the spatiotemporal treatment of trajectory data. One can, for example, construct a pairwise distance matrix, calculated across all time steps, that can be used as input for the clustering algorithms. Moreover, certain distance (similarity) measures can account for small local variations in speed or temporal offset between trajectories,^79^ as discussed in the following subsection.

With the aim of this paper being to demonstrate an approach for identifying concentrations of reaction flux that is both straightforward and that can be performed at low computational expense, we focus on the time-series treatment of trajectory data and mark the more involved spatiotemporal approach as something that could be considered in future work. We also note that although deep-learning approaches for identifying temporal patterns in trajectory data exist,^80^ we focus on unsupervised algorithms that do not require a complicated training phase and thus can be quickly deployed in an almost automatic fashion to aid the interpretation of simulation and experimental observables.^81,82^

### 2.4 Computational Details

Prior to clustering, the selected features must be normalized since their numerical magnitude may vary significantly. For example, in our application, the dihedral angles span a significantly larger range of values than the C_1_–C_6_ bond length. The normalization thus limits the risk that features which span a larger numerical range dominate the clustering results. Here, we utilize the so-called min–max normalization, in which we map each feature to the range [0, 1] as
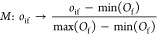
1where *o*_if_ refers to the trajectory *o*_i_ along the feature dimension f and *o*_if_ ∈ *O*_f_, where *O*_f_ is the set of all trajectories across feature f. With the normalized data, we proceed to construct a pairwise distance matrix, where each element corresponds to a single scalar value that reflects the similarity of the corresponding pair of trajectories, considering all features included (in the current example four features).

**Figure 5:**
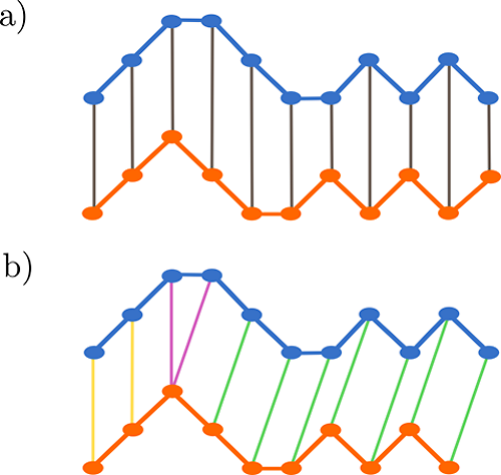
Mapping of two time series, shown in blue and orange. (a) Direct 1:1 mapping, as in the *L*_2_ norm, (b) DTW allows 1:many mapping, allowing similar sequences to be mapped onto each other even when not perfectly aligned. In panel (b), the different possible mappings of temporal indices are indicated by their color, with yellow representing a 1:1 mapping of equivalent temporal indices, magenta a 1:many mapping, and green the warping of the temporal axis through a 1:1 mapping between indices at different time steps.

The choice of distance and similarity measure depends on the data. In this paper, we consider the multidimensional generalization of the *L*_*p*_ norm and multidimensional dynamic time warping (MD-DTW).^79,83^ Starting with the *L*_*p*_ norm, this considers the distance between each sequential point in the time series and involves a 1:1 mapping of time points, as demonstrated in Figure 5a. The multidimensional generalization of the *L*_*p*_ norm for two trajectories *o*_*i*_ and *o*_*j*_ is defined as
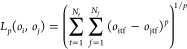
2with *N*_*t*_ the total number of time steps and *N*_*f*_ the total numbers of features. In the following, we exclusively use *p* = 2, i.e., the *L*_2_ norm which is the generalization of the Euclidean distance. The advantage of this norm is its conceptual and computational simplicity. A drawback is that the *L*_*p*_ norms require that both trajectories are the same length in time and therefore might not be applicable to trajectory data where the duration of trajectories varies (for instance, if trajectories are not propagated beyond fragmentation). Moreover, *L*_*p*_ norms have been shown to underperform on high-dimensional data.^84^ In addition, the 1:1 mapping of time points is insufficiently flexible to account for small variations on the time axis. For example, two trajectories may be highly similar apart from a slight offset in time or exhibit a small local variation in frequency. In some cases, this may result in the misclassification of trajectories as the full extent of their similarity is not captured.

Some of these shortcomings can be overcome by dynamic time warping (DTW),^85,86^ which is the second similarity measure that we evaluate. This was originally developed for applications in speech recognition.^85^ Since then, it has been employed in a wide range of fields and applied to many different types of time-series data.^15,70,73,87−89^ In brief, DTW allows for a degree of stretching or contraction on the time axis. This enables 1:many mapping of temporal indices, as illustrated in Figure 5b, meaning one can compare two time series that are similar but have local variations in frequency and temporal offsets. Furthermore, this makes it possible to compare time-series data of different lengths. Note that depending on the implementation, DTW may be fully frequency invariant, which could lead to erroneous matching of different vibrational frequencies. However, it is possible to constrict the degree to which this happens. As such, we favor limiting the amount of frequency invariance and primarily suggest the use of DTW to account for small temporal offsets between similar trajectories, as outlined in the Supporting Information.

The mapping of temporal indices is given by the warping path, which describes how a pair of trajectories are warped in time so that they are optimally aligned. The warping path is calculated by tracing through a cost matrix, which defines all the possible ways the two time series can be warped in time. Further details on how the warping path is calculated from the cost matrix and the dynamic programming procedure can be found in the Supporting Information. Once the warping path is calculated, the total cost of alignment is given by the sum of all its elements, which serves as the measure of distance between two trajectories.

While a plethora of clustering algorithms exist, including agglomerative-,^90^ centroid-,^91^ and probabilistic^92^-based methods, we choose to focus on density-based approaches.^93^ Generally, these aim to assign data points to clusters based on the local density of points within a given volume. The definition of density varies between methods. In the case of DBSCAN,^94^ which is the method explored in this paper, core regions of density are built up by identifying regions where a minimum number of data points (given by the parameter MinPts) lie within a given radius (ϵ) of each other. We focus on DBSCAN due to its ability to detect nonconvex clusters without requiring the user to specify a predefined number clusters. Furthermore, DBSCAN inherently includes outlier detection, marking data points that do not lie in sufficiently dense regions of the feature space, defined by MinPts and ϵ, as noise. This makes it less sensitive to outlier data points that do not correspond to the main trends in the dataset. An additional benefit of the DBSCAN algorithm is that it can be easily modified to accept precomputed pairwise distance matrices as input. Constructing a distance matrix using both the multidimensional *L*_2_ norm and MD-DTW, the whole time series can be clustered globally over all time. A more detailed discussion of the DBSCAN algorithm and the types of clusters that may be identified is provided in the Supporting Information.

Having run the DBSCAN algorithm, one obtains *N*_C_ clusters, with *C*_I_ the number of members in cluster *I* ∈ *N*_C_. The validity of the clustering can be assessed by considering the number of trajectories classified as noise *N*_noise_^a^ and the silhouette coefficient, which provides a statistical measure of how well a trajectory is assigned to its cluster. The silhouette coefficient *S*_*i*_ for trajectory *o*_*i*_ in cluster *I* is defined as^95^
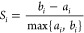
3This has two components. The *a*_*i*_ term is the mean distance between trajectory *o*_*i*_ and all other trajectories within its cluster *I*,
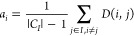
4where |*C*_*I*_| is the number of members in cluster I. A small value of *a*_*i*_ therefore means that trajectory *o*_*i*_ is well assigned to cluster I. The *b*_*i*_ in eq 3, on the other hand, is the mean distance of trajectory *o*_*i*_ to the trajectories in its nearest-neighbor cluster *J*,
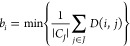
5where the min{} function is used to identify the nearest neighbor among all clusters *J* ∈ *N*_C_. A large value of *b*_*i*_ indicates that trajectory *i* is distinct from the trajectories in the nearest-neighbor cluster.

Together *a*_*i*_ and *b*_*i*_ define the silhouette coefficient *S*_*i*_, whose values are in the range *S*_*i*_ ∈ [−1, 1]. The closer *S*_*i*_ is 1, the more appropriately the trajectory is clustered. However, if *S*_*i*_ < 0, then trajectory *o*_*i*_ would be more appropriately assigned to its nearest-neighboring cluster. The average value of the silhouette coefficient over all clustered data points (*S*_avg_) provides a global measure that can be used to assess the validity of the clustering.

## 3 Results and Discussion

We shall now turn to a discussion of the results obtained by the clustering of the data set consisting of CHD trajectories presented in Section 2.1 using the four features as shown in Figure 3 (i.e., the three dihedral angles ϕ_a_, ϕ_b_, and ϕ_c_ and the *R*_C1–C6_ bond length). The similarity of every trajectory pair is calculated using both the *L*_2_ norm and MD-DTW as discussed in Section 2.4. The resulting distance matrix is then provided as a precalculated input into the DBSCAN algorithm. In each case, we scan a suitable range of possible ϵ and MinPts values and analyze the clustering results.

### 3.1 Multidimensional L_2_ Norm

**Figure 6:**
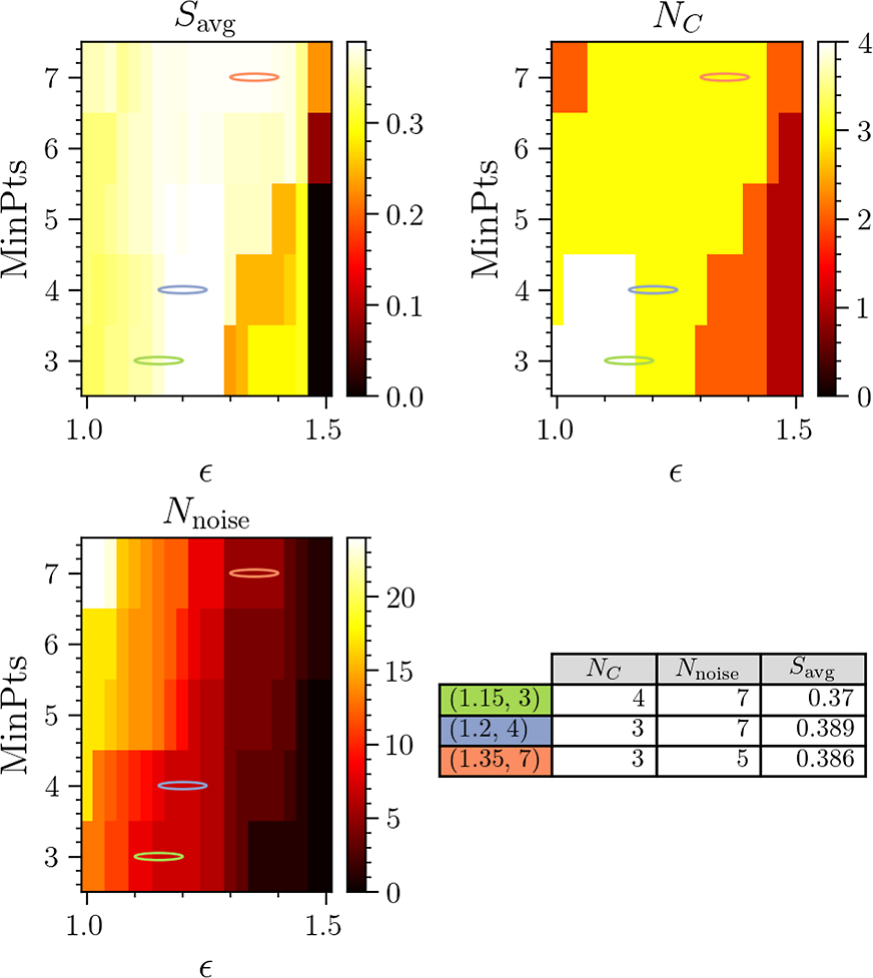
Results of clustering using the *L*_2_ norm as a measure of trajectory similarity. The heatmaps shown correspond to (top left) the average silhouette coefficient *S*_avg_, (top right) the number of resulting clusters *N*_C_, and (bottom left) the total number of trajectories marked as noise *N*_noise_. Note that the range of the heatmap for *S*_avg_ does not reflect the full [−1, 1] range available to *S*_avg_ in order to visualize the variations correctly. The table (bottom right) shows the values of *N*_C_, *N*_noise_, and *S*_avg_ for the good clustering results with *N*_C_ = 3, 4, encircled by correspondingly colored ellipses in the heatmaps.

With the L_2_ norm as a similarity measure, the parameters ϵ and MinPts are scanned in the ranges ϵ ∈ [1, 1.5] and MinPts ∈ [3, 7], respectively, repeating the clustering for each pair of parameters. The results are presented in Figure 6, which shows the number of clusters *N*_C_, the average value of the silhouette coefficient *S*_avg_, and the number of trajectories marked as noise *N*_noise_. Good clustering is reflected in large values of the average silhouette coefficient *S*_avg_. Generally, the highest values of *S*_avg_ result in either *N*_C_ = 3 or *N*_C_ = 4 clusters being identified. Note, for many combinations of ϵ and MinPts, there is little variation in the value of *S*_avg_, making the identification of the best set of parameters, and thus also the ideal number of clusters, somewhat ambiguous. The best combinations of parameter values are indicated by the circled points in the heatmaps, with the corresponding values given in the table in Figure 6.

Overall, the region corresponding to ϵ > 1.3 and MinPts < 6 yields the smallest values of *S*_avg_. For these parameter values, the definition of the density neighborhood is too diffuse, and trajectories are grouped into only *N*_C_ ≤ 2 broad clusters. Increasing the value of MinPts to ≥6 raises the density requirement, enabling *N*_C_ = 3 clusters to be identified. In this range, the best results are given by (ϵ, MinPts) = (1.35, 7), circled in orange in the heatmaps in Figure 6. As seen in the table in Figure 6, these parameters yield *N*_noise_ = 5 and *S*_avg_ = 0.386. Moreover, the intermediate range of ϵ, where 1.175 ≤ ϵ ≤ 1.3, yields *N*_C_ = 3 with the highest value of *S*_avg_ across all values of MinPts, indicating optimal cluster separability. However the number of trajectories marked as noise increases above 10 for high values of MinPts. The best *N*_C_ = 3 case is given by (ϵ, MinPts) = (1.2, 4) and is circled by a blue oval in each panel in Figure 6. In this case, only seven trajectories are marked as noise, with a slightly higher value of *S*_avg_ = 0.389 compared to (ϵ, MinPts) = (1.35, 7). Finally, when ϵ ≤ 1.15, the density neighborhood is sufficiently small to identify *N*_C_ = 4 clusters, but with as many as 15–20 trajectories marked as noise. However, picking a lower value of MinPts = 3 yields reasonable levels of noise. For this particular case, (ϵ, MinPts) = (1.15, 3), *N*_C_ = 4 clusters are identified with only seven noise trajectories (green oval in Figure 6). This yields *S*_avg_ = 0.37, which is slightly lower than the *N*_C_ = 3 region.

**Figure 7:**
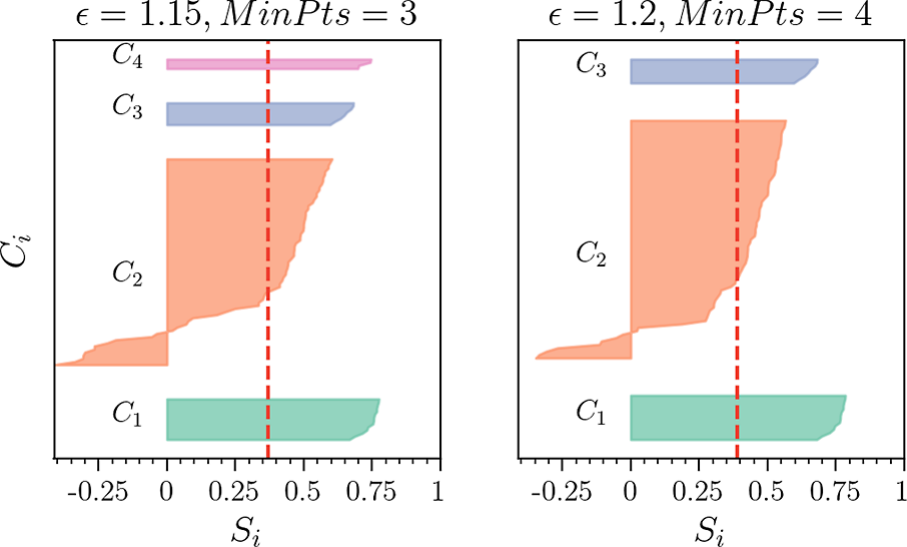
Values of the silhouette coefficients (*S*_*i*_) for each trajectory in each cluster *C*_I_, with the whole area from zero to *S*_*i*_ filled by the color of each cluster. The left panel shows results for (ϵ, MinPts) = (1.15, 3) and the right for (ϵ, MinPts) = (1.2, 4). The height of the shaded area reflects the size of each cluster. The two sets of parameters for which results are shown correspond to the best results for *N*_C_ = 3 and 4, in each case with seven trajectories excluded as noise. The red dashed lines indicate the average silhouette coefficient, *S*_avg_.

**Figure 8:**
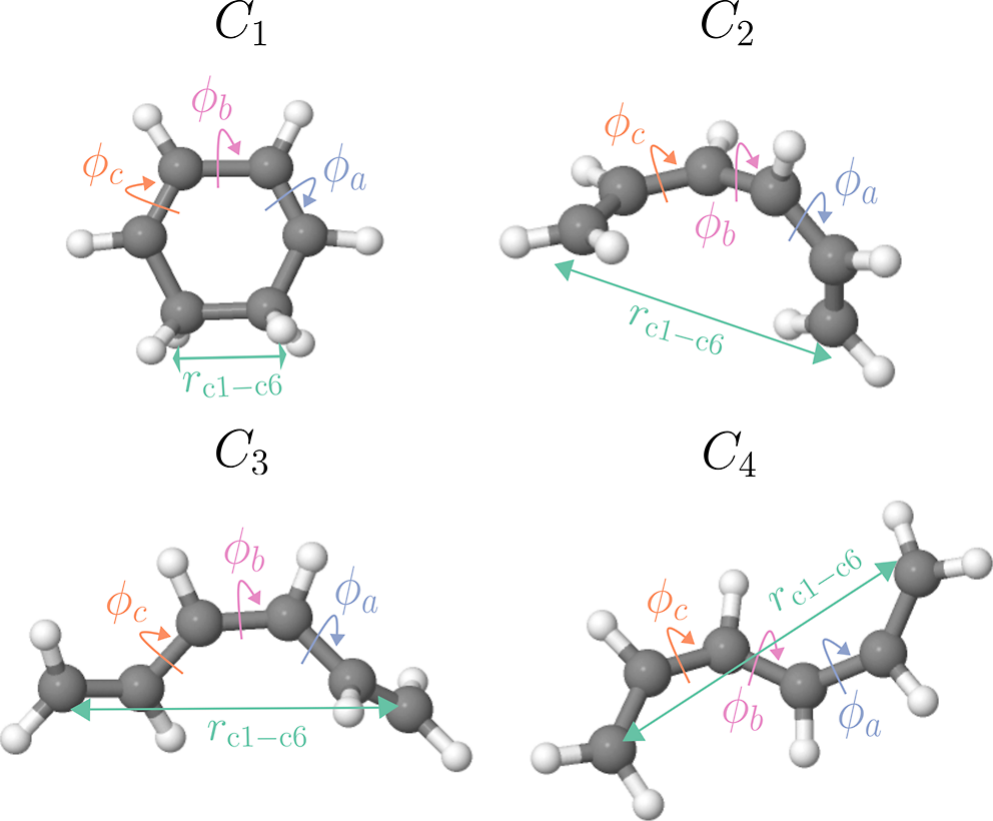
Set of four product channels identified by clustering. Shown are the final geometries at the center of each cluster. *C*_1_ corresponds to the return of the wave packet to hot CHD, *C*_2_ to the part of the wave packet that remains on the excited state and exhibits larger torsional angles, and *C*_3_ to the ground-state tZc/tZt HT product. Finally, *C*_4_ corresponds to a cEc-HT product.

To better understand the clustering results, we turn to Figure 7, which shows the values of the individual silhouette coefficients *S*_*i*_ for each trajectory in its respective cluster *C*_*i*_. Looking first at the (ϵ, MinPts) = (1.15, 3) case in the left-hand panel, we notice that the *S*_*i*_ values of *C*_1_, *C*_3_, and *C*_4_ are all above the average of *S*_avg_ = 0.37, indicating that the trajectories therein are well clustered. However, the broad shaded area corresponding to *C*_2_ indicates the existence of a large cluster that contains some trajectories for which *S*_*i*_ < *S*_avg_. In addition, *C*_2_ contains a number of trajectories for which *S*_*i*_ < 0, meaning that they are unfavorably clustered and would in principle be better assigned to the nearest-neighboring cluster instead. Turning to the (ϵ, MinPts) = (1.2, 4) case in the right panel of Figure 7, we see that cluster *C*_4_ is absorbed by cluster *C*_2_. However, the number of trajectories with *S*_*i*_ < 0 is reduced. This, and the slight increase in *S*_svg_ from 0.37 to 0.389, indicates that (ϵ, MinPts) = (1.2, 4) results in a marginally improved clustering. The reaction products identified by the center of each cluster at the final time are shown in Figure 8. For the *N*_C_ = 3 case, the products correspond to vibrationally hot CHD (*C*_1_), a set of structures with large torsional angles (*C*_2_), and tZt-HT (*C*_3_), which is formed via isomerization of cZc-HT. The *N*_C_ = 4 case results in the identification of an additional product (*C*_4_), corresponding to cEc-HT.

**Figure 9:**
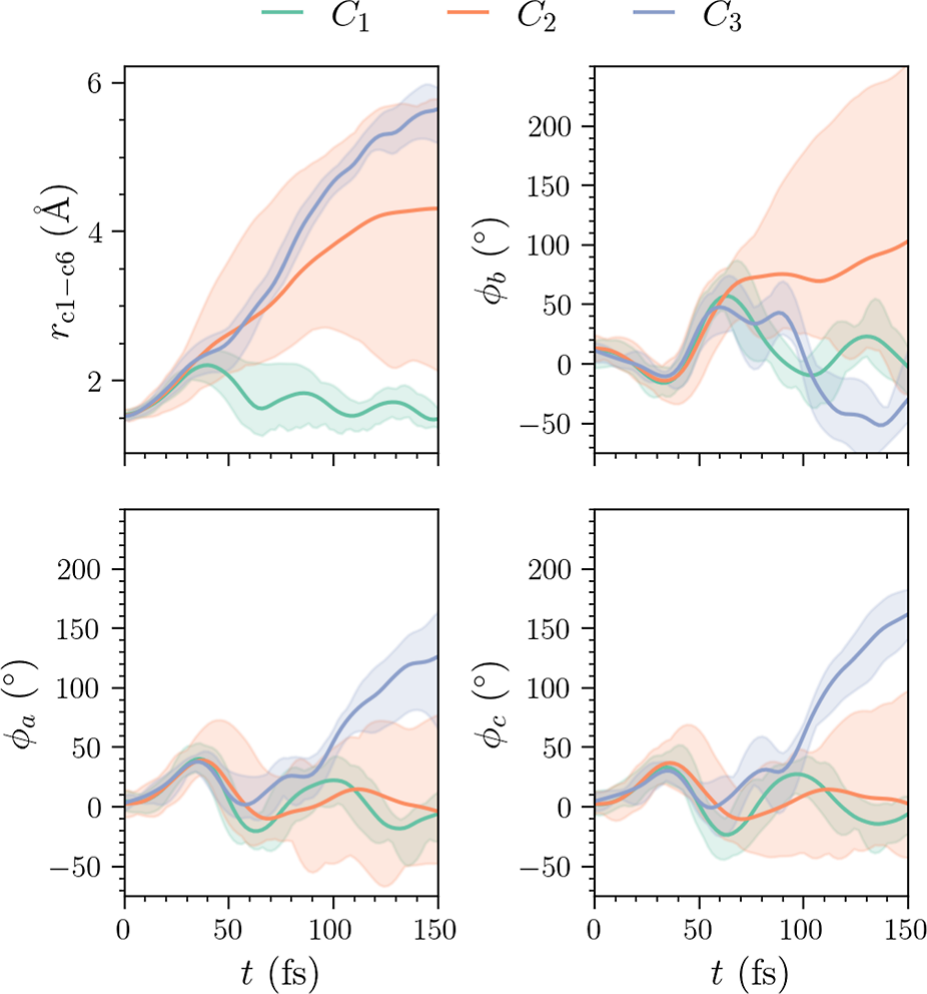
Plots of the cluster centers in each of the four selected features for ϵ = 1.2 and MinPts = 4 (using the *L*_2_ norm). The top left and right panels show the C_1_–*C*_6_ bond and the central dihedral angle ϕ_b_, whereas the bottom left and right panels show the dihedral angles ϕ_a_ and ϕ_c_. The shaded areas represent the size of the cluster.

Having identified the *N*_C_ = 3 case with (ϵ, MinPts) = (1.2, 4) as the statistically better result, although marginally so, we now turn to discuss the distribution and evolution of the clusters in time. The resulting clusters *C*_1_ through to *C*_3_ can be seen in Figure 9, where each panel refers to one of the four features on which clustering was performed. Before approximately 40 fs, all clusters exhibit a similar evolution, with the CHD ring structure strained upon excitation. The strained geometry is indicated by the increase in the dihedral angles ϕ_a_ and ϕ_c_, as well as the C_1_–C_6_ bond length. The straining of the ring system continues, with oscillations in ϕ_a_ and ϕ_c_ and a simultaneous sharp increase in the dihedral ϕ_b_. Following this, the cluster centers begin to separate as the trajectories disperse, evolving along different reaction pathways. Cluster *C*_1_ returns to vibrationally hot CHD after several oscillations in the C_1_–C_6_ distance, which dampen in time. Turning to cluster *C*_3_, we observe that the C_1_–C_6_ bond length increases at a faster rate than the other clusters. This occurs as cZc-HT forms on the ground state. For times exceeding 100 fs, *C*_3_ begins to increase in both the ϕ_a_ and ϕ_c_ dihedral angles, commensurate with the isomerization of cZc-HT to tZt-HT. Characterizing the pathway for cluster *C*_2_ is less clear-cut, with the cluster exhibiting significant broadening in all four features. This broadening is most significant for the C_1_–C_6_ distance and ϕ_b_ dihedral, which span almost the whole range of available distances and angles. This is likely the result of a very disperse wave packet limiting the separability of the data, at least based on nuclear geometries alone. However, we do note that the cluster is centered on the values of ϕ_b_ around 100° and ϕ_a_/ϕ_c_ around zero degrees. Thus, the densest component of *C*_2_ corresponds to the part of the excited wave packet that experiences dispersion along the ϕ_b_ coordinate, resulting in a greater degree of torsion as the trajectories remain trapped on the flat part of the *S*_1_ state.

**Figure 10:**
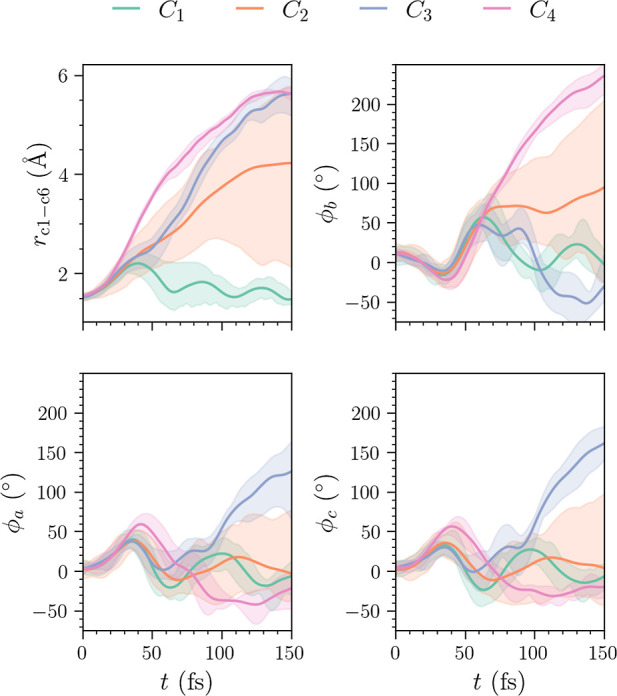
Plots of the cluster centers in each of the four selected features for ϵ = 1.15 and MinPts = 3 (using the *L*_2_ norm). The top left and right panels show the C_1_–C_6_ bond and the central dihedral angle ϕ_b_, whereas the bottom left and right panels show the dihedral angles ϕ_a_ and ϕ_c_. The shaded areas represent the size of the cluster.

Turning to the *N*_C_ = 4 case with (ϵ, MinPts) = (1.15, 3), plotted in Figure 10, we observe the same general trends as shown in Figure 9. However, we see that some trajectories are redistributed from the broad *C*_2_ cluster to a new cluster *C*_4_. While the dihedral angles ϕ_b_ still span a large range, this is somewhat reduced. Recall that here cluster *C*_2_ contains a greater number of trajectories with negative values of the silhouette coefficient *S*_*i*_, as shown in Figure 7. It is, therefore, likely that the very broad nature of the wave packet here results in clusters that have a degree of overlap and that some trajectories within *C*_2_ are misclassified. The additional cluster *C*_4_ observes very small values of the dihedral angles ϕ_a_/ϕ_c_. In addition, the sharper increase in the C_1_–C_6_ bond length, and the fact the dihedral angle ϕ_b_ exceeds 200°, suggests this cluster is commensurate with the cEc-HT product channel. Here, we note that both ϕ_a_ and ϕ_c_ exhibit strong oscillations before the central dihedral ϕ_b_ rapidly increases, suggesting that the initial ring strain transfers energy to the dihedral ϕ_b_.

**Figure 11:**
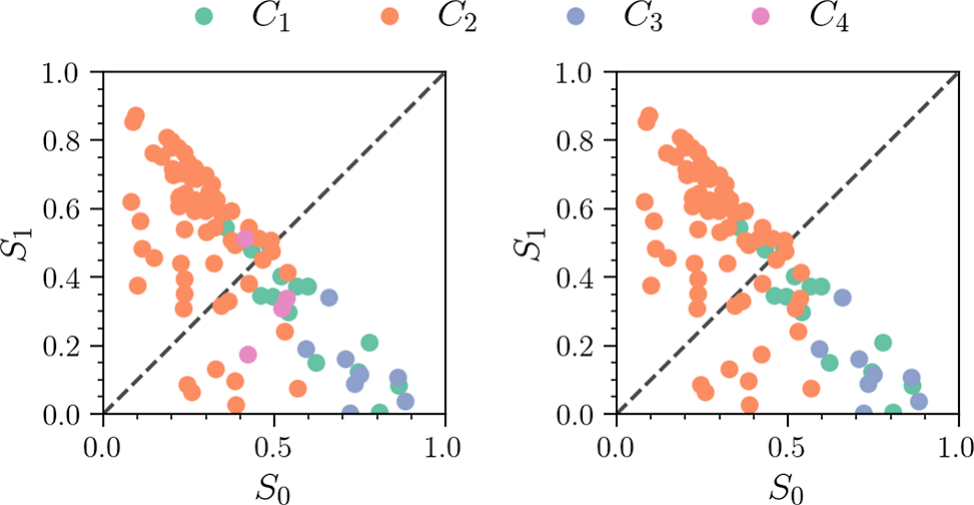
Distribution of *S*_0_ and *S*_1_ state populations at 150 fs. The left panel shows the *N*_C_ = 4 resulting from parameters (ϵ, MinPts) = (1.15, 3), and the right panel shows the *N*_C_ = 3 case with (ϵ, MinPts) = (1.2, 4).

We now shift our focus to the electronic-state populations for each cluster. The populations are not included in the feature set in the current clustering process and provide additional insight into the nature of each identified cluster. In Figure 11 we see the populations of the *S*_1_ and *S*_0_ state plotted for every trajectory in each cluster at 150 fs. The dashed line *x* = *y* is drawn to indicate that trajectories below and above this line are mostly *S*_0_ and *S*_1_ in character, respectively. Moreover, the left and right hand panels show the *N*_C_ = 4 and *N*_C_ = 3 clustering results, respectively. In both panels, we see that clusters *C*_1_ and *C*_3_ are mostly *S*_0_ in character, confirming that they correspond to the return of the wave packet to ground-state CHD and the ground-state HT product channel that isomerizes from cZc-HT to cZt- and tZt-HT. However, the character of *C*_2_ is again less clear. While most trajectories are dominated by *S*_1_ population, some trajectories have higher *S*_0_ population. This supports the idea that the densest part of *C*_2_ defines the part of the wave packet that remains trapped on the excited state, dispersing into a broader range of ϕ_b_ angles due to the low gradients on the PES. It also suggests that some of the trajectories are clustered unfavorably, a fact also supported by the negative *S*_*i*_ values. In the left-hand panel, we see that in the *N*_C_ = 4 case, some of the *C*_2_ trajectories on the ground state are relabeled as *C*_4_, corresponding to cEc-HT.

### 3.2 Multidimensional Dynamic Time Warping

To improve on the issue with some misclassified *C*_2_ trajectories when using the *L*_2_ norm, we turn to MD-DTW as a more flexible similarity measure. Given that MD-DTW can account for small variations in offsets between trajectories and small local changes in frequency, it has the potential to provide a more accurate measure of similarity between the temporal series, thus improving separability compared to when the *L*_2_ norm is used.

**Figure 12:**
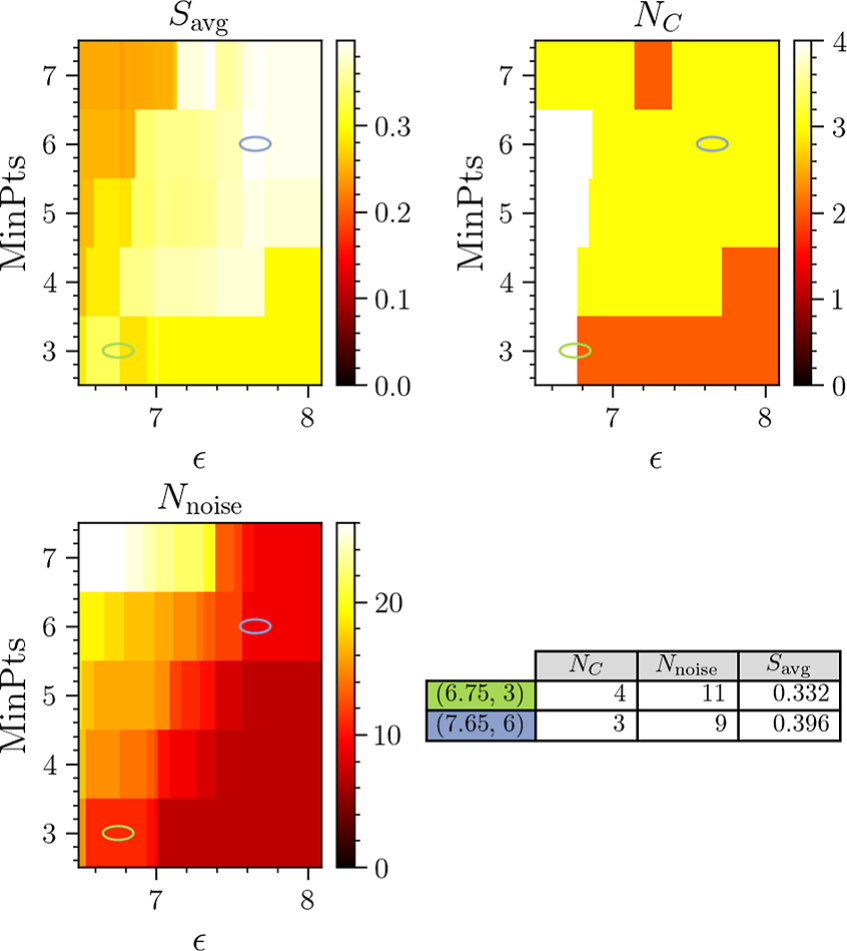
Results of clustering when using DTW as a measure of trajectory similarity. The scale of the *S*_avg_ heatmap is chosen to highlight the differences in otherwise small variations. The table shows data for the two best clustering results with *N*_C_ = 3 and 4, highlighted by colored ellipses in the heatmaps. The corresponding figure for the *L*_2_ norm is Figure 6.

Here, the parameters ϵ and MinPts are scanned in the ranges ϵ ∈ [6.5, 8] and MinPts ∈ [3, 7], respectively. The result of the clustering is shown in Figure 12 in terms of *N*_C_, *N*_noise_, and *S*_avg_. Generally, performing DBSCAN clustering using the MD-DTW similarity measure yields similar results to using the *L*_2_ norm, with either three or four clusters identified. The highest values of *S*_avg_ are observed as MinPts increases and ϵ lies in the range 7.6 ≤ ϵ ≤ 8. This results in *N*_C_ = 3 clusters. The highest value of *S*_avg_ = 0.396 corresponds to (ϵ, MinPts) = (7.65, 6), indicated by the purple ellipse in each heatmap. The intermediate range where 6.9 ≤ ϵ ≤ 7.6 consistently results in *N*_C_ = 3 clusters. The higher values of MinPts result in an excessive number of trajectories marked as noise. However, the values of ϵ ≤ 7.7 and MinPts ≤ 6 consistently result in *N*_C_ = 4 clusters. The number of trajectories excluded as noise increases with MinPts. In addition, the value of *S*_avg_ decreases slightly in comparison with the *N*_C_ = 3 results. The set of values (ϵ, MinPts) = (6.75, 3) results in *N*_C_ = 4 clusters with lower noise levels and a reasonable value of *S*_avg_ = 0.332, and is indicated by the green ellipse in the heatmaps.

**Figure 13:**
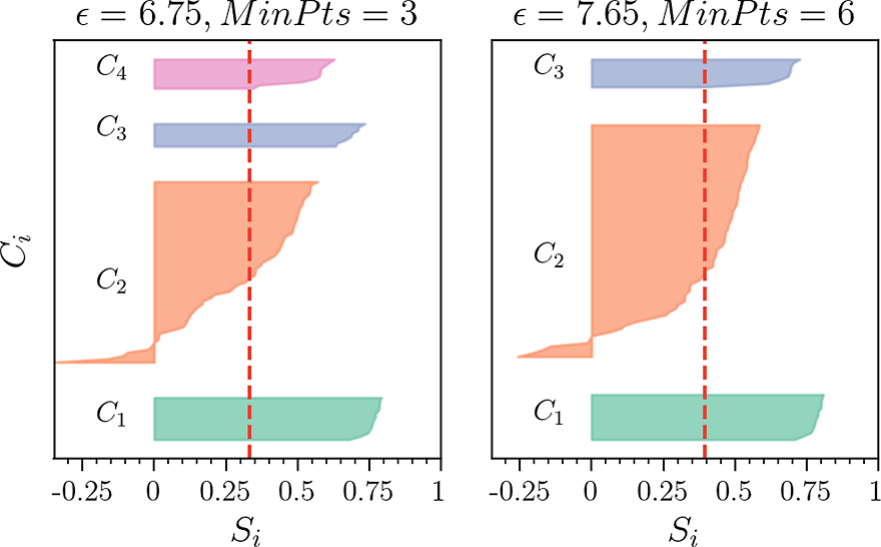
Silhouette coefficient (*S*_*i*_) for each trajectory in its respective cluster *C*_*i*_ (using MD-DTW). The left-hand panel shows the best *N*_C_ = 4 set of parameters, and the right-hand panel, the best *N*_C_ = 3 case. In each case, the red dashed line indicates the value of *S*_avg_. The corresponding figure for the *L*_2_ norm is Figure 7.

Turning to Figure 13, which shows for the best *N*_C_ = 3 and 4 cases the silhouette coefficient *S*_*i*_ for each trajectory in its respective cluster, we see that the *C*_4_ cluster is significantly larger in comparison with the *N*_C_ = 4 case in Figure 7. Here, additional trajectories are acquired from the *C*_2_ cluster. Furthermore, *C*_2_ appears to have a smaller number of trajectories with negative *S*_*i*_ values. For the set of parameters (ϵ, MinPts) = (7.65, 6), which has the highest *S*_avg_, the *C*_4_ cluster is again absorbed by the *C*_2_ cluster. Note that apart from a small number of trajectories, the *N*_C_ = 3 case is practically indistinguishable from the corresponding *N*_C_ = 3 results identified by the *L*_2_ norm. However, the *N*_C_ = 4 case, as identified by MD-DTW, is unique. We shall, therefore, focus the rest of our discussion on this result, noting that the *C*_4_ cluster is always absorbed by the *C*_2_ cluster in the set of *N*_C_ = 3 results.

**Figure 14:**
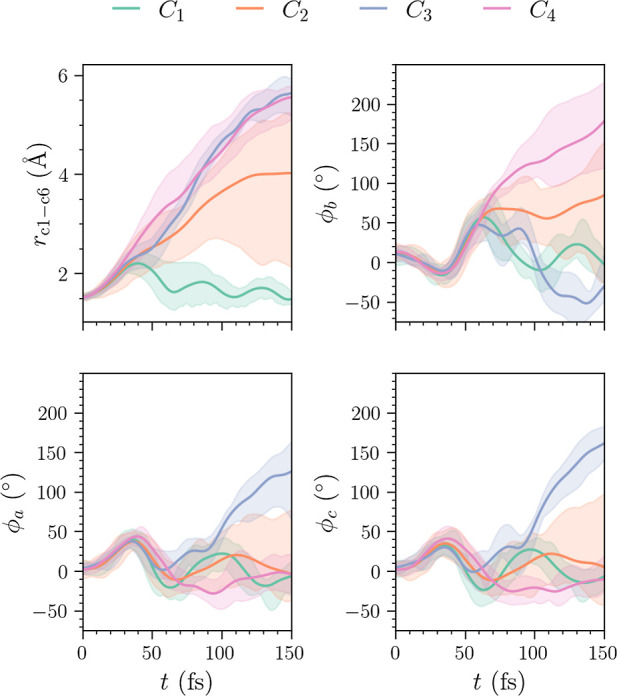
Plots of the cluster centers in each of the four selected features for ϵ = 6.75 and MinPts = 3 (using MD-DTW). The top left and right panels show the C_1_–C_6_ bond and the central dihedral angle ϕ_b_, whereas the bottom left and right panels show the dihedral angles ϕ_a_ and ϕ_c_. The shaded areas represent the size of the cluster.

The plot of the features for the center of each cluster in the *N*_C_ = 4 case is shown in Figure 14. The *C*_4_ cluster now includes a broader range of dihedral angles ϕ_b_ in comparison with the *L*_2_ norm case in Figure 10. At the end of time, this cluster is centered at a slightly lower value of ϕ_b_ (170°). However, it still contains trajectories that exceed angles ϕ_b_ > 200°. Also note that the trajectories with lower values of ϕ_b_ come from the *C*_2_ cluster, which is now slightly smaller than in the *L*_2_ norm case. The broad range of dihedral angles ϕ_b_ makes separation of these clusters difficult. Based on nuclear geometries alone, it is not immediately clear if additional trajectories in *C*_4_ correspond to ground-state cEc-HT. Without additional information, the separation of the two groups of trajectories, corresponding to ground-state cEc-HT and the remainder of the excited wave packet with larger torsional angles, may not be possible. Analogously to the *L*_2_ norm as shown in Figure 9, the *N*_C_ = 3 case appears by clusters *C*_2_ and *C*_4_ in Figure 14 merging to form a very broad and large cluster *C*_2_.

**Figure 15:**
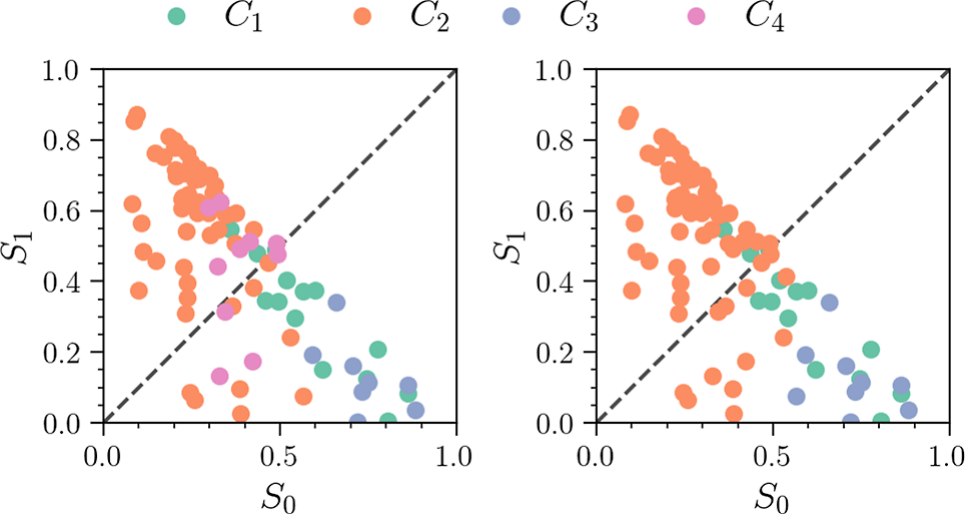
Distribution of *S*_0_ and *S*_1_ state populations at time *t* = 150 fs using MD-DTW. Points are color-coded according to their cluster assignment. The left panel shows the *N*_C_ = 4 resulting from (ϵ, MinPts) = (6.75, 3), and the right panel shows the *N*_C_ = 3 case with (ϵ, MinPts) = (7.65, 6).

Turning to Figure 15, which shows the final-time (*t* = 150 fs) distribution of populations observed within each cluster, we see that the *N*_C_ = 3 case (right panel) is nearly identical to the *L*_2_ norm results as shown in Figure 11. In the left panel, we see the *N*_C_ = 4 result that is unique to MD-DTW. Here, we see a number of additional *C*_4_ trajectories with a population above the *x* = *y* line. Thus, the additional trajectories contained within the *C*_4_ cluster are dominated by the *S*_1_ state amplitude. These trajectories belong to the remainder of the excited wave packet that exhibits a larger degree of torsion and not cEc-HT formation on the ground state. However, we note that these trajectories have a population that is nearly equal for the *S*_0_ and *S*_1_ states. Were the simulations to run longer, these may completely decay to the ground state and yield the cEc-HT product. Moreover, we see that while the *C*_2_ cluster is composed mostly of trajectories with dominant *S*_1_ character, it still contains a number of trajectories with dominant *S*_0_ character.

## 4 Conclusions

The approach demonstrated in this paper is general and applicable to any trajectory-based simulation method. It is easy to implement, computationally efficient, and provides a useful tool that can be deployed with little additional effort for quick identification of key reaction and product channels. In addition, once the clusters have been obtained, a small set of representative trajectories can be identified that can be used for, e.g., high-level calculations of observables.

Notably, the clustering is applied to the entire basis, and the weights of the individual trajectories are not considered at the clustering stage. While the size of each resulting cluster gives some indication of the significance of the corresponding reactive pathway, in the case of e.g. the AIMCE trajectories considered in this paper, a more accurate measure would involve integration over the complex expansion coefficients of the trajectories, as outlined in the Supporting Information.

Generally, the clusters identified have a degree of overlap and vary in both size and density. In part, the difficulty in achieving good separation of the data relates to the limited ability of DBSCAN to identify clusters of varying density, a known issue, and that we have applied the algorithm to a test system in which the wave packet at later times is quite disperse. Nevertheless, given the nature of the trajectory data and that the time-series approach requires an algorithm capable of utilizing precomputed distance matrices, DBSCAN is the best choice. Other algorithms that accept precomputed distance matrices, such as *k*-means or hierarchical clustering, are generally not suitable and in testing we found them to yield noninterpretable and nonsensical results, although we note that this observation may to some degree be system-dependent.

The time-series methodology does have drawbacks. First, the similarity measures are global over time, which means that smaller amplitude motions at earlier times can wash out in systems that exhibit large amplitude motion at later times, such as CHD. The clustering results are, in those cases, biased toward later times, where the system has settled into a distribution of different products. This is likely the cause of the similar results obtained with both the multidimensional *L*_2_ norm and the MD-DTW. As such, the potential benefit of using MD-DTW does not outweigh the additional computation expense, and we favor the use of the more simplistic *L*_2_ norm in systems with large amplitude motion. For both similarity measures, we see that based on nuclear geometries alone, the time-series approach can struggle to separate trajectories contained in a broad distribution of dihedral angles. Although MD-DTW achieves better separation, it still misclassifies some trajectories. On a positive note, both metrics succeed in identifying four separate reaction channels and provide basic insight into the decay pathways available to the excited system.

Second, consider the scenario where a large swarm of trajectories decay through a CoIn, leaving a number of trajectories behind that undergo several additional oscillations on the excited state before decaying. The straggling trajectories slowly leak through the CoIn, before converging on the product. Given that the separation of trajectories in the time-series approach depends largely on the time at which a significant number of trajectories assume a different geometry, it is only able to capture the large swarm trajectories that decay over a similar time period. The use of MD-DTW may compensate for this to a greater extent than the *L*_2_ norm due to its ability to account for offsets in time. However, in our current example, the choice of similarity metric had little effect on the clustering result. Finally, for very long simulations, the use of MD-DTW is not practical due to its unfavorable scaling with the length of the temporal series, although restrictions may be placed on the number of warping paths considered, thus increasing efficiency. The multidimensional L_2_ norm is also limited by its poor performance in high-dimensional spaces.

In the application to CHD, we demonstrate that a suitable set of four features can be selected from the internal coordinate based on their high variance. In systems that exhibit a wider and more complex range of nuclear rearrangements, more features may have to be included in the clustering. Generally, increasing the number of features also increases the sampling requirement, and therefore, the results have a dependence on the number of trajectories and the underlying statistical distribution of the data. If the sampling of phase space is insufficient, there is a risk that minor pathways will be marked as outliers. Thus, sufficient sampling of the phase space is just as important for accurate clustering, as it is for overall accurate propagation of the dynamics. In terms of features, in some cases, the rearrangement of the nuclear geometry may be sufficiently complex to warrant alternative representations of the nuclear motion beyond internal coordinates. This could include constructing coordinates or displacement vectors that capture the coupled simultaneous motion of several atoms. Future work should, therefore, address the limits of internal coordinates and the construction of alternative coordinates. Furthermore, the use of more sophisticated approaches that construct reduced dimensionality representations of the dynamics poses an interesting avenue for further exploration. For instance, one may envision the use of autoencoders to encode the time-dependent trends of the dynamics in a reduced latent space.

While the current work allows for a rapid first-order identification of different reaction channels available, it cannot describe more intricate details of the nuclear and electronic dynamics observed. Future work to be undertaken should, therefore, focus on methods that provide more in-depth insights into complex trajectory datasets, likely by exploring the applicability of the more demanding spatiotemporal treatment described in Section 2.3. Due to the large overlap of trajectories within the Franck–Condon region at early times and the subsequent emergence of different reaction pathways with variable probability density, the difficulty lies in achieving good clustering over all times without having to reparameterize the chosen clustering algorithm at each independent time step. Should such an approach be successfully realized, this may be capable of not only identifying the different reaction pathways but also the times at which the trajectories diverge. Furthermore, one could envision the inclusion of additional information in the clustering process, such as momenta or electronic populations, allowing for improved refinement of the key trends observed and providing insight into the changing electronic character during the evolution of the system.

In summary, we have demonstrated an automatic and unbiased computational procedure for harvesting physical and chemical insights from large sets of high-dimensional and highly complex trajectories. We have shown that clustering using the unsupervised density-based DBSCAN algorithm provides a computationally efficient method for classifying trajectories, allowing the main trends in the probability flux to be rapidly identified at low computational cost. This methodology slots naturally into the standard workflow for simulations of photoexcited dynamics that employ trajectory-based (quantum) molecular dynamics methods. Compared to deep-learning approaches, this method requires no complicated training phase and can be deployed in an almost automatic fashion to aid the interpretation of simulations and experimental observables.

## Supporting Information Available

Background on the AIMCE simulations used to calculate the trajectories, an overview of types of clusters and clustering algorithms, and further details on the identification or reactive pathways and dynamic time warping (PDF)

## Footnotes

a The corresponding percent is *P*_noise_ = 100 × *N*_noise_/*N*_trj_, with *N*_trj_ the total number of trajectories. Since *N*_trj_ = 100, the conversion is trivial for current data.

## References

[ref1] Stolow A.; Bragg A. E.; Neumark D. M. Femtosecond Time-Resolved Photoelectron Spectroscopy. Chem. Rev. 2004, 104, 1719–1758. 10.1021/cr020683w. 15080710

[ref2] Ischenko A. A.; Weber P. M.; Miller R. J. D. Capturing Chemistry in Action with Electrons: Realization of Atomically Resolved Reaction Dynamics. Chem. Rev. 2017, 117, 11066–11124. 10.1021/acs.chemrev.6b00770. 28590727

[ref3] Amini K.; Biegert J. Ultrafast electron diffraction imaging of gas-phase molecules. Advances In Atomic, Molecular, and Optical Physics; Elseiver, 2020; Vol. 69, pp 163–231.

[ref4] Stankus B.; Yong H.; Ruddock J.; Ma L.; Carrascosa A. M.; Goff N.; Boutet S.; Xu X.; Zotev N.; Kirrander A.; Minitti M.; Weber P. M. Advances in Ultrafast Gas-Phase X-ray Scattering. J. Phys., B 2020, 53, 234004 10.1088/1361-6455/abbfea.

[ref5] Rolles D. Time-resolved experiments on gas-phase atoms and molecules with XUV and X-ray free-electron lasers. Adv. Phys.: X 2023, 8, 2132182 10.1080/23746149.2022.2132182.

[ref6] Mai S.; Marquetand P.; González L. Quantum Chemistry and Dynamics of Excited States: Methods and Applications, 1st ed.; González L., Lindh R., Eds.; John Wiley and Sons: UK, 2020; Chapter 16, p 499.

[ref7] Curchod B. F. E. Quantum Chemistry and Dynamics of Excited States: Methods and Applications, 1st ed.; González L., Lindh R., Eds.; John Wiley and Sons: UK, 2020; Chapter 14, p 435.

[ref8] Makhov D. V.; Symonds C.; Fernandez-Alberti S.; Shalashilin D. V. Ab initio quantum direct dynamics simulations of ultrafast photochemistry with Multiconfigurational Ehrenfest approach. Chem. Phys. 2017, 493, 200–218. 10.1016/j.chemphys.2017.04.003.

[ref9] Kirrander A.; Vacher M. Quantum Chemistry and Dynamics of Excited States: Methods and Applications, 1st ed.; González L., Lindh R., Eds.; John Wiley and Sons: UK, 2020; Chapter 15, p 469.

[ref10] Worth G. A.; Lasorne B. Quantum Chemistry and Dynamics of Excited States: Methods and Applications, 1st ed.; González L., Lindh R., Eds.; John Wiley and Sons: UK, 2020; Chapter 13, p 413.

[ref11] Westermayr J.; Gastegger M.; Schütt K. T.; Maurer R. J. Perspective on integrating machine learning into computational chemistry and materials science. J. Chem. Phys. 2021, 154, 230903 10.1063/5.0047760. 34241249

[ref12] Shi Y.; Wang D.; Tang J.; Deng M.; Liu H.; Liu B. Detecting spatiotemporal extents of traffic congestion: a density-based moving object clustering approach. Int. J. Geogr. Inf. Sci. 2021, 35, 1449–1473. 10.1080/13658816.2021.1905820.

[ref13] Si Y.; Skidmore A. K.; Wang T.; De Boer W. F.; Debba P.; Toxopeus A. G.; Li L.; Prins H. H.; Prins H. H. T. Spatio-temporal dynamics of global H5N1 outbreaks match bird migration patterns. Geospatial Health 2009, 4, 65–78. 10.4081/gh.2009.211. 19908191

[ref14] Yang P.; Dumont G.; Ansermino J. Adaptive Change Detection in Heart Rate Trend Monitoring in Anesthetized Children. IEEE Trans. Biomed. 2006, 53, 2211–2219. 10.1109/tbme.2006.877107. 17073326

[ref15] Gupta K.; Chatterjee N. Financial Time Series Clustering. Information and Communication Technology for Intelligent Systems; ICTIS 2017: Cham, 2018; Vol. 2, pp 146–156.

[ref16] Acheson K.; Kirrander A. Robust Inversion of Time-Resolved Data via Forward-Optimization in a Trajectory Basis. J. Chem. Theory Comput. 2023, 19, 2721–2734. 10.1021/acs.jctc.2c01113. 37129988 PMC10210245

[ref17] Yong H.; Moreno Carrascosa A.; Ma L.; Stankus B.; Minitti M. P.; Kirrander A.; Weber P. M. Determination of excited state molecular structures from time-resolved gas-phase X-ray scattering. Faraday Discuss 2021, 228, 104–122. 10.1039/d0fd00118j. 33595043

[ref18] Asenov M.; Ramamoorthy S.; Zotev N.; Kirrander A. Inversion of Ultrafast X-ray Scattering with Dynamics Constraints. Machine Learning and the Physical Sciences, 2020; p 7.

[ref19] Yang J.; Zandi O.; Zhang P.; Centurion M. Imaging of molecules in the gas phase with ultrafast electron diffraction. Ultrafast Nonlinear Imaging and Spectroscopy II, 2014; Vol. 9198, pp 100–110.

[ref20] Hensley C. J.; Yang J.; Centurion M. Imaging of Isolated Molecules with Ultrafast Electron Pulses. Phys. Rev. Lett. 2012, 109, 133202 10.1103/physrevlett.109.133202. 23030087

[ref21] Habershon S.; Zewail A. H. Determining Molecular Structures and Conformations Directly from Electron Diffraction using a Genetic Algorithm. Chem. Phys. Chem. 2006, 7, 353–362. 10.1002/cphc.200500532. 16411250

[ref22] Kirrander A.; Saita K.; Shalashilin D. V. Ultrafast X-ray Scattering from Molecules. J. Chem. Theory Comput. 2016, 12, 957–967. 10.1021/acs.jctc.5b01042. 26717255

[ref23] Stefanou M.; Saita K.; Shalashilin D. V.; Kirrander A. Comparison of Ultrafast Electron and X-Ray Diffraction – A Computational Study. Chem. Phys. Lett. 2017, 683, 300–305. 10.1016/j.cplett.2017.03.007.

[ref24] Oana C. M.; Krylov A. I. Dyson orbitals for ionization from the ground and electronically excited states within equation-of-motion coupled-cluster formalism: Theory, implementation, and examples. J. Chem. Phys. 2007, 127, 234106 10.1063/1.2805393. 18154374

[ref25] Northey T.; Zotev N.; Kirrander A. *Ab Initio* Calculation of Molecular Diffraction. J. Chem. Theory Comput. 2014, 10, 4911–4920. 10.1021/ct500096r. 26584376

[ref26] Northey T.; Kirrander A. Ab Initio Fragment Method for Calculating Molecular X-ray Diffraction. J. Phys. Chem. A 2019, 123, 3395–3406. 10.1021/acs.jpca.9b00621. 30892904

[ref27] Moreno Carrascosa A.; Yong H.; Crittenden D. L.; Weber P. M.; Kirrander A. Ab-initio calculation of total x-ray scattering from molecules. J. Chem. Theor. Comput. 2019, 15, 2836–2846. 10.1021/acs.jctc.9b00056. 30875212

[ref28] Zotev N.; Moreno Carrascosa A.; Simmermacher M.; Kirrander A. Excited Electronic States in Total Isotropic Scattering from Molecules. J. Chem. Theory Comput. 2020, 16, 2594–2605. 10.1021/acs.jctc.9b00670. 32142278

[ref29] Mašín Z.; Benda J.; Gorfinkiel J. D.; Harvey A. G.; Tennyson J. UKRmol+: A suite for modelling electronic processes in molecules interacting with electrons, positrons and photons using the R-matrix method. Comput. Phys. Commun. 2020, 249, 107092 10.1016/j.cpc.2019.107092.

[ref30] Tudorovskaya M.; Minns R. S.; Kirrander A. Effects of probe energy and competing pathways on time-resolved photoelectron spectroscopy: the ring-opening of 1,3-cyclohexadiene. Phys. Chem. Chem. Phys. 2018, 20, 17714–17726. 10.1039/c8cp02397b. 29876567

[ref31] Lange O. F.; Grubmüller H. Full correlation analysis of conformational protein dynamics. Proteins 2007, 70, 1294–1312. 10.1002/prot.21618. 17876828

[ref32] Li W.; Fu L.; Niu B.; Wu S.; Wooley J. Ultrafast clustering algorithms for metagenomic sequence analysis. Briefings Bioinf. 2012, 13, 656–668. 10.1093/bib/bbs035. PMC3504929 22772836

[ref33] Foy S. G.; Wilson B. A.; Bertram J.; Cordes M. H. J.; Masel J. A Shift in Aggregation Avoidance Strategy Marks a Long-Term Direction to Protein Evolution. Genetics 2019, 211, 1345–1355. 10.1534/genetics.118.301719. 30692195 PMC6456324

[ref34] Braun J.; Fayne D. Mapping of Protein Binding Sites using clustering algorithms - Development of a pharmacophore based drug discovery tool. J. Mol. Graph. Model. 2022, 115, 108228 10.1016/j.jmgm.2022.108228. 35667141

[ref35] Audagnotto M.; Czechtizky W.; De Maria L.; Käck H.; Papoian G.; Tornberg L.; Tyrchan C.; Ulander J. Machine learning/molecular dynamic protein structure prediction approach to investigate the protein conformational ensemble. Sci. Rep. 2022, 12, 10018 10.1038/s41598-022-13714-z. 35705565 PMC9200820

[ref36] Aggarwal C. C.; Reddy C. K. Data Clustering: Algorithms and Applications; Chapman and Hall/CRC, 2016.

[ref37] Minitti M.; Budarz J.; Kirrander A.; Robinson J.; Ratner D.; Lane T.; Zhu D.; Glownia J.; Kozina M.; Lemke H.; Sikorski M.; Feng Y.; Nelson S.; Saita K.; Stankus B.; Northey T.; Hastings J.; Weber P. Imaging Molecular Motion: Femtosecond X-Ray Scattering of an Electrocyclic Chemical Reaction. Phys. Rev. Lett. 2015, 114, 255501 10.1103/physrevlett.114.255501. 26197134

[ref38] Woodward R. B.; Hoffmann R. Stereochemistry of Electrocyclic Reactions. J. Am. Chem. Soc. 1965, 87, 395–397. 10.1021/ja01080a054.

[ref39] Hoffmann R.; Woodward R. B. Conservation of orbital symmetry. Acc. Chem. Res. 1968, 1, 17–22. 10.1021/ar50001a003.

[ref40] Woodward R. B.; Hoffmann R. The Conservation of Orbital Symmetry. Angew. Chem., Int. Ed. 1969, 8, 781–853. 10.1002/anie.196907811.

[ref41] Garavelli M.; Celani P.; Fato M.; Bearpark M. J.; Smith B. R.; Olivucci M.; Robb M. A. Relaxation Paths from a Conical Intersection: The Mechanism of Product Formation in the Cyclohexadiene/Hexatriene Photochemical Interconversion. J. Phys. Chem. A 1997, 101, 2023–2032. 10.1021/jp961554k.

[ref42] Fuß W.; Schmid W. E.; Trushin S. A. Time-resolved dissociative intense-laser field ionization for probing dynamics: Femtosecond photochemical ring opening of 1,3-cyclohexadiene. J. Chem. Phys. 2000, 112, 8347–8362. 10.1063/1.481478.

[ref43] Hofmann A.; de Vivie-Riedle R. Quantum dynamics of photoexcited cyclohexadiene introducing reactive coordinates. J. Chem. Phys. 2000, 112, 5054–5059. 10.1063/1.481059.

[ref44] Ruan C.-Y.; Lobastov V.; Srinivasan R.; Goodson B.; Ihee H.; Zewail A. Ultrafast diffraction and structural dynamics: The nature of complex molecules far from equilibrium. Proc. Natl. Acad. Sci. U.S.A. 2001, 98, 7117–7122. 10.1073/pnas.131192898. 11404473 PMC34632

[ref45] Dudek R. C.; Weber P. M. Ultrafast Diffraction Imaging of the Electrocyclic Ring-Opening Reaction of 1,3-Cyclohexadiene. J. Phys. Chem. A 2001, 105, 4167–4171. 10.1021/jp010122t.

[ref46] Garavelli M.; Page C. S.; Celani P.; Olivucci M.; Schmid W. E.; Trushin S. A.; Fuss W. Reaction Path of a sub-200 fs Photochemical Electrocyclic Reaction. J. Phys. Chem. A 2001, 105, 4458–4469. 10.1021/jp010359p.

[ref47] Tamura H.; Nanbu S.; Nakamura H.; Ishida T. A theoretical study of cyclohexadiene/hexatriene photochemical interconversion: multireference configuration interaction potential energy surfaces and transition probabilities for the radiationless decays. Chem. Phys. Lett. 2005, 401, 487–491. 10.1016/j.cplett.2004.11.111.

[ref48] Tamura H.; Nanbu S.; Ishida T.; Nakamura H. Ab initio nonadiabatic quantum dynamics of cyclohexadiene/hexatriene ultrafast photoisomerization. J. Chem. Phys. 2006, 124, 084313 10.1063/1.2171688. 16512722

[ref49] Kosma K.; Trushin S. A.; Fuß W.; Schmid W. E. Cyclohexadiene ring opening observed with 13 fs resolution: coherent oscillations confirm the reaction path. Phys. Chem. Chem. Phys. 2009, 11, 172–181. 10.1039/b814201g. 19081921

[ref50] Schönborn J. B.; Sielk J.; Hartke B. Photochemical Ring-Opening of Cyclohexadiene: Quantum Wavepacket Dynamics on a Global Ab Initio Potential Energy Surface. J. Phys. Chem. A 2010, 114, 4036–4044. 10.1021/jp909362c. 20210344

[ref51] Deb S.; Weber P. M. The Ultrafast Pathway of Photon-Induced Electrocyclic Ring-Opening Reactions: The Case of 1,3-Cyclohexadiene. Annu. Rev. Phys. Chem. 2011, 62, 19–39. 10.1146/annurev.physchem.012809.103350. 21054174

[ref52] Bühler C. C.; Minitti M. P.; Deb S.; Bao J.; Weber P. M. Ultrafast Dynamics of 1,3-Cyclohexadiene in Highly Excited States. J. Phys. B: At., Mol. Opt. Phys. 2011, 2011, 1–6. 10.1155/2011/637593.

[ref53] Minitti M. P.; Budarz J. M.; Kirrander A.; Robinson J.; Lane T. J.; Ratner D.; Saita K.; Northey T.; Stankus B.; Cofer-Shabica V.; Hastings J.; Weber P. M. Toward structural femtosecond chemical dynamics: imaging chemistry in space and time. Faraday Discuss. 2014, 171, 81–91. 10.1039/c4fd00030g. 25415842

[ref54] Adachi S.; Sato M.; Suzuki T. Direct Observation of Ground-State Product Formation in a 1,3-Cyclohexadiene Ring-Opening Reaction. J. Phys. Chem. Lett. 2015, 6, 343–346. 10.1021/jz502487r. 26261944

[ref55] Pemberton C. C.; Zhang Y.; Saita K.; Kirrander A.; Weber P. M. From the (1B) Spectroscopic State to the Photochemical Product of the Ultrafast Ring-Opening of 1,3-Cyclohexadiene: A Spectral Observation of the Complete Reaction Path. J. Phys. Chem. A 2015, 119, 8832–8845. 10.1021/acs.jpca.5b05672. 26192201

[ref56] Budarz J. M.; Minitti M. P.; Cofer-Shabica D. V.; Stankus B.; Kirrander A.; Hastings J. B.; Weber P. M. Observation of Femtosecond Molecular Dynamics via Pump-probe Gas Phase X-Ray Scattering. J. Phys., B 2016, 49, 034001 10.1088/0953-4075/49/3/034001.

[ref57] Ruddock J. M.; Yong H.; Stankus B.; Du W.; Goff N.; Chang Y.; Odate A.; Carrascosa A. M.; Bellshaw D.; Zotev N.; Liang M.; Carbajo S.; Koglin J.; Robinson J. S.; Boutet S.; Kirrander A.; Minitti M. P.; Weber P. M. A deep UV trigger for ground-state ring-opening dynamics of 1,3-cyclohexadiene. Sci. Adv. 2019, 5, eaax6625 10.1126/sciadv.aax6625. 31523713 PMC6731073

[ref58] Wolf T. J. A.; Sanchez D. M.; Yang J.; Parrish R. M.; Nunes J. P. F.; Centurion M.; Coffee R.; Cryan J. P.; Gühr M.; Hegazy K.; Kirrander A.; Li R. K.; Ruddock J.; Shen X.; Vecchione T.; Weathersby S. P.; Weber P. M.; Wilkin K.; Yong H.; Zheng Q.; Wang X. J.; Minitti M. P.; Martínez T. J. The photochemical ring-opening of 1,3-cyclohexadiene imaged by ultrafast electron diffraction. Nat. Chem. 2019, 11, 504–509. 10.1038/s41557-019-0252-7. 30988415

[ref59] Polyak I.; Hutton L.; Crespo-Otero R.; Barbatti M.; Knowles P. J. Ultrafast Photoinduced Dynamics of 1,3-Cyclohexadiene Using XMS-CASPT2 Surface Hopping. J. Chem. Theory Comput. 2019, 15, 3929–3940. 10.1021/acs.jctc.9b00396. 31244132

[ref60] Filatov M.; Lee S.; Nakata H.; Choi C. H. Structural or population dynamics: what is revealed by the time-resolved photoelectron spectroscopy of 1,3-cyclohexadiene? A study with an ensemble density functional theory method. Phys. Chem. Chem. Phys. 2020, 22, 17567–17573. 10.1039/d0cp02963g. 32716454

[ref61] Yong H.; Zotev N.; Ruddock J. M.; Stankus B.; Simmermacher M.; Carrascosa A. M.; Du W.; Goff N.; Chang Y.; Bellshaw D.; Liang M.; Carbajo S.; Koglin J. E.; Robinson J. S.; Boutet S.; Minitti M. P.; Kirrander A.; Weber P. M. Observation of the molecular response to light upon photoexcitation. Nat. Commun. 2020, 11, 2157 10.1038/s41467-020-15680-4. 32358535 PMC7195484

[ref62] Karashima S.; Humeniuk A.; Uenishi R.; Horio T.; Kanno M.; Ohta T.; Nishitani J.; Mitrić R.; Suzuki T. Ultrafast Ring-Opening Reaction of 1,3-Cyclohexadiene: Identification of Nonadiabatic Pathway via Doubly Excited State. J. Am. Chem. Soc. 2021, 143, 8034–8045. 10.1021/jacs.1c01896. 34027664

[ref63] Guerra C.; Ayarde-Henríquez L.; Duque-Noreña M.; Chamorro E. On Electron Pair Rearrangements in Photochemical Reactions: 1,3-Cyclohexadiene Ring Opening. J. Phys. Chem. A 2022, 126, 395–405. 10.1021/acs.jpca.1c07800. 34923827

[ref64] Travnikova O.; Piteša T.; Ponzi A.; Sapunar M.; Squibb R. J.; Richter R.; Finetti P.; Di Fraia M.; De Fanis A.; Mahne N.; Manfredda M.; Zhaunerchyk V.; Marchenko T.; Guillemin R.; Journel L.; Prince K. C.; Callegari C.; Simon M.; Feifel R.; Decleva P.; Došlić N.; Piancastelli M. N. Photochemical Ring-Opening Reaction of 1,3-Cyclohexadiene: Identifying the True Reactive State. J. Am. Chem. Soc. 2022, 144, 21878–21886. 10.1021/jacs.2c06296. 36444673 PMC9732879

[ref65] Huan L.; Tang J.; Alelyani S. Data Clustering; Chapman and Hall/CRC, 2014.

[ref66] Jolliffe I. T. Principal Component Analysis. Springer Series in Statistics; Springer-Verlag: New York, 2002.

[ref67] Hinton G. E.; Salakhutdinov R. R. Reducing the Dimensionality of Data with Neural Networks. Science 2006, 313, 504–507. 10.1126/science.1127647. 16873662

[ref68] Olive X.; Basora L.; Viry B.; Alligier R. Deep Trajectory Clustering with Autoencoders. 9th International Conference for Research in Air Transportation, 2020.

[ref69] Lim K.-L.; Jiang X.; Yi C. Deep Clustering With Variational Autoencoder. IEEE Signal Process. Lett. 2020, 27, 231–235. 10.1109/lsp.2020.2965328.

[ref70] Li K.; Sward K.; Deng H.; Morrison J.; Habre R.; Franklin M.; Chiang Y.-Y.; Ambite J. L.; Wilson J. P.; Eckel S. P. Using dynamic time warping self-organizing maps to characterize diurnal patterns in environmental exposures. Sci. Rep. 2021, 11, 24052 10.1038/s41598-021-03515-1. 34912034 PMC8674322

[ref71] Tseng K.-K.; Li J.; Tang Y.-J.; Yang C.-W.; Lin F.-Y.; Zhao Z. Clustering Analysis of Aging Diseases and Chronic Habits With Multivariate Time Series Electrocardiogram and Medical Records. Front. Aging Neurosci. 2020, 12, 10.3389/fnagi.2020.00095. PMC7232580 32477093

[ref72] Goutte C.; Toft P.; Rostrup E.; Nielsen F. A.; Hansen L. K. On Clustering fMRI Time Series. Neuroimage 1999, 9, 298–310. 10.1006/nimg.1998.0391. 10075900

[ref73] Aghabozorgi S.; Seyed Shirkhorshidi A.; Ying Wah T. Time-series clustering—A decade review. Inf. Syst. 2015, 53, 16–38. 10.1016/j.is.2015.04.007.

[ref74] Warren Liao T. Clustering of time series data—a survey. Pattern Recogn. 2005, 38, 1857–1874. 10.1016/j.patcog.2005.01.025.

[ref75] Krumm J. Computing with Spatial Trajectories; Zheng Y., Zhou X., Eds.; Springer: New York, NY, 2011; pp 213–241.

[ref76] Zhu W.; Chen J.; Xu L.; Cao J. Recognition of interactive human groups from mobile sensing data. Comput. Commun. 2022, 191, 208–216. 10.1016/j.comcom.2022.04.028.

[ref77] Jeung H.; Yiu M. L.; Jensen C. S. Computing with Spatial Trajectories; Zheng Y., Zhou X., Eds.; Springer, 2011; pp 143–177.

[ref78] Kisilevich S.; Mansmann F.; Nanni M.; Rinzivillo S. Data Mining and Knowledge Discovery Handbook; Maimon O., Rokach L., Eds.; Springer: United States, 2010; pp 855–874.

[ref79] Tao Y.; Both A.; Silveira R. I.; Buchin K.; Sijben S.; Purves R. S.; Laube P.; Peng D.; Toohey K.; Duckham M. A comparative analysis of trajectory similarity measures. GIScience Remote Sens. 2021, 58, 643–669. 10.1080/15481603.2021.1908927.

[ref80] Goodfellow I.; Yoshua B.; Courville A. Deep Learning; The MIT Press: Cambridge, 2017.

[ref81] Gabalski I.; Sere M. A.; Acheson K.; Allum F.; Boutet S.; Dixit G.; Forbes R.; Glownia J. M.; Goff N.; Hegazy K.; Howard A. J.; Liang M.; Minitti M. P.; Minns R. S.; Natan A.; Peard N.; Rasmus W. O.; Sension R. J.; Ware M. R.; Weber P. M.; Werby N.; Wolf T. J. A.; Kirrander A.; Bucksbaum P. Transient vibration and product formation of photoexcited CS2 measured by time-resolved X-ray scattering. J. Chem. Phys. 2022, 157, 164305 10.1063/5.0113079. 36319419 PMC9625835

[ref82] Razmus W. O.; Acheson K.; Bucksbaum P.; Centurion M.; Champenois E.; Gabalski I.; Hoffman M. C.; Howard A.; Lin M.-F.; Liu Y.; Nunes P.; Saha S.; Shen X.; Ware M.; Warne E. M.; Weinacht T.; Wilkin K.; Yang J.; Wolf T. J. A.; Kirrander A.; Minns R. S.; Forbes R. Multichannel photodissociation dynamics in CS2 studied by ultrafast electron diffraction. Phys. Chem. Chem. Phys. 2022, 24, 15416–15427. 10.1039/d2cp01268e. 35707953

[ref83] Kotsakos D.; Gunopulos D.; Trajcevski G.; Aggarwal C. C. Data Clustering; Chapman and Hall/CRC, 2014.

[ref84] Aggarwal C. C.; Hinneburg A.; Keim D. A. Database Theory—ICDT 2001; Springer Berlin Heidelberg, 2001; Vol. 1973; pp 420–434.

[ref85] Sakoe H.; Chiba S. Dynamic programming algorithm optimization for spoken word recognition. IEEE Trans. Acoust. Speech Signal Process. 1978, 26, 43–49. 10.1109/tassp.1978.1163055.

[ref86] Giorgino T. Computing and Visualizing Dynamic Time Warping Alignments in R: The dtw Package. J. Stat. Software 2009, 31, 1–24. 10.18637/jss.v031.i07.

[ref87] Dupas R.; Tavenard R.; Fovet O.; Gilliet N.; Grimaldi C.; Gascuel-Odoux C. Identifying seasonal patterns of phosphorus storm dynamics with dynamic time warping. Water Resour. Res. 2015, 51, 8868–8882. 10.1002/2015wr017338.

[ref88] Chen M. Time Series Clustering and Classification. J. Am. Stat. Assoc. 2020, 115, 1558 10.1080/01621459.2020.1801281.

[ref89] Javed A.; Lee B. S.; Rizzo D. M. A benchmark study on time series clustering. Mach. Learn. Appl. 2020, 1, 100001 10.1016/j.mlwa.2020.100001.

[ref90] Nielsen F. Introduction to HPC with MPI for Data Science; Nielsen F., Ed.; Undergraduate Topics in Computer Science; Springer International Publishing: Cham, 2016; pp 195–211.

[ref91] Jin X.; Han J. Encyclopedia of Machine Learning and Data Mining; Sammut C., Webb G. I., Eds.; Springer US: Boston, MA, 2017; pp 695–697.

[ref92] Jiawei H.; Deng H. Data Clustering; Chapman and Hall/CRC, 2014; Chapter 3, pp 61–86.

[ref93] Ester M. Data Clustering; Chapman and Hall/CRC, 2014; Chapter 5, pp 111–126.

[ref94] Ester M.; Kriegel H.; Sander J.; Xu X. A Density-Based Algorithm for Discovering Clusters in Large Spatial Databases with Noise; Knowledge Discovery and Data Mining, 1996.

[ref95] Rousseeuw P. J. Silhouettes: A graphical aid to the interpretation and validation of cluster analysis. J. Comput. Appl. Math. 1987, 20, 53–65. 10.1016/0377-0427(87)90125-7.

