# Supplementary material for: Automatic Clustering of Excited-State Trajectories: Application to Photoexcited Dynamics
Source: J Chem Theory Comput. 2023 Sep 13;19(18):6126–38. doi: 10.1021/acs.jctc.3c00776 (PMC10536988; doi:10.1021/acs.jctc.3c00776)
Supplement: Supplementary file 1 — ct3c00776_si_001.pdf [file ct3c00776_si_001.pdf]

Supplementary Information: Automatic Clustering of Excited-State Trajectories: Application to Photoexcited Dynamics

Kyle Acheson[†,¶] and Adam Kirrander[*,‡]

†*EaStCHEM, School of Chemistry and Centre for Science at Extreme Conditions, University of Edinburgh, David Brewster Road, Edinburgh EH9 3FJ, United Kingdom*

‡*Physical and Theoretical Chemistry Laboratory, Department of Chemistry, University of Oxford, Oxford OX1 3QZ, United Kingdom*

¶*Department of Chemistry, University of Warwick, Coventry CV4 7AL, United Kingdom*

E-mail: adam.kirrander@chem.ox.ac.uk

Phone: +(0)1865 275422. Fax: +(0)1865 275400

1 Trajectory Simulations

The set of 100 trajectories that model the electrocyclic ring opening of 1,3-cyclohexadiene (CHD) to 1,3,5-hexatriene (HT), are simulated using the *ab-initio* multiconfigurational Ehrenfest (AIMCE) method. The total molecular wavefunction is expanded in a basis of N_{traj} Ehrenfest wavepackets[1–4],

$$|\Psi(t)\rangle = \sum_{n=1}^{N_{\text{traj}}} c_n(t)\, |\psi_n(t)\rangle\,, \tag{S1}$$

with each Ehrenfest wavepacket,

$$|\psi_n(t)\rangle = \left[\sum_{\alpha=1}^{N_s} a_\alpha^n(t)\, |\psi_\alpha\rangle\right] |\chi_n(t)\rangle\,, \tag{S2}$$

and the nuclear wavepacket $|\chi_n(t)\rangle$ defined as a Gaussian in a harmonic potential,

$$|\chi_n(t)\rangle = \left(\frac{\sigma}{\pi}\right)^{N_d/4} \exp\left(-\frac{\sigma}{2}\left(\mathbf{R}-\mathbf{R}_n(t)\right)^2 + \frac{i}{\hbar}\mathbf{P}_n(t)\left(\mathbf{R}-\mathbf{R}_n(t)\right) + \frac{i}{\hbar}\gamma_n(t)\right), \tag{S3}$$

where $\mathbf{R}_n$ and $\mathbf{P}_n$ are the position and momentum coordinates at the centre of the Gaussian, $\mathbf{R}$ the set of nuclear coordinates over the width of the Gaussian, and γ_n the phase of the Gaussian. The Gaussian has dimensionality N_d, corresponding to the number of degrees of freedom of the system. Each of the widths σ are assumed fixed, and are specified for each individual degree of freedom according to the type of atom. The coefficients $a_\alpha^n(t)$ in Eq. S2 correspond to the contribution of each quantum state α to its respective Ehrenfest wavepacket. These are normalised to unity for each wavepacket and $|a_\alpha(t)|^2$ gives the electronic population of state α at time t. The transfer of population between electronic states is therefore modelled as a change in the amplitude of the coefficients $a_\alpha^n(t)$. Note, the coefficients $a_\alpha^n(t)$ are not coupled between each of the n trajectories and each individual Ehrenfest wavepacket is propagated independently according to Ehrenfest equations of motion, guided by the phase space coordinates at the centre of the Gaussian. The Ehrenfest wavepacket's are coupled a posteriori through the complex coefficients $c_n(t)$ in Eq. S1, which account for the contribution of each wavepacket to the molecular wavefunction[3,4]. In the

sudden approximation, the coefficients $c_n(t)$ are initialised as $1/N_{\mathrm{traj}}$. However, if the simulations include an explicit excitation pulse, the initial values of the coefficients are given by the excitation. While there exist formulations of MCE in which the evolution of the Ehrenfest wavepackets are coupled at all time steps, this is not conducive to *ab-initio* direct dynamics.

Each Ehrenfest wavepacket $|\chi_n(t)\rangle$, defined by Eq. S2, follows phase space coordinates $(\mathbf{R}_n(t), \mathbf{P}_n(t))$, and are propogated according to the equation of motion,

$$F_n = -\sum_{\alpha=1}^{N_s} |a_\alpha^n(t)|^2 \frac{dE_\alpha}{d\mathbf{R}_n} - \sum_{\alpha\neq\beta}^{N_s} a_\alpha^{n*}(t)\, a_\beta^n(t)\, \mathbf{d}_{\alpha\beta}\left(E_\beta - E_\alpha\right), \tag{S4}$$

where the first term represents the gradient of the electronic states weighted by their respective populations, meaning that the nuclei evolve on the potential energy surfaces (PESs) in a mean-field fashion. The second term corresponds to the gradient in the direction of the non-adiabatic coupling matrix elements (NACMEs), and thus it accounts for changes in the force due to nonadiabatic population transfer. In the mean-field treatment of the force, the larger contributions are therefore from the states with the most significant population at a given point in time.

The time evolution of each Ehrenfest wavepackets coefficients a_α^n, is defined by,

$$\dot{a}_\alpha^n = -\frac{i}{\hbar}\sum_{\beta=1}^{N_s} \langle\chi_n(t)|\langle\psi_\alpha|\left[\hat{H}_e + \hat{T}_n\right]|\psi_\beta\rangle|\chi_n(t)\rangle\, a_\beta^n(t) - \left\langle \chi_n \middle| \frac{\partial\chi_n}{\partial t}\right\rangle a_\alpha^n(t), \tag{S5}$$

where the total Hamiltonian has been split into its electronic $\hat{H}_e$ and nuclear kinetic energy $\hat{T}_n$ terms. For an in-depth discussion of the contributions to this equation from the matrix elements on the left hand side, as well as how the equation is solved, can be found in ref. 5.

The complex coupling coefficients $c_n(t)$ (Eq. S1) that couple each Ehrenfest wavepacket, are propagated according to the equation of motion,

$$\sum_n \Omega_{mn}\dot{c}_n(t) = -i\left[\langle\psi_m(t)|\hat{H}|\psi_n(t)\rangle - i\hbar\left\langle \psi_m(t) \middle| \frac{\partial\psi_n(t)}{\partial t}\right\rangle\right] c_n(t), \tag{S6}$$

where $\Omega_{mn} = \langle\psi_n(t)|\psi_m(t)\rangle$ - i.e. the overlap of two Ehrenfest wavepackets, and $\dot{c}_n(t)$ refers to

the partial derivative of $c_n(t)$ with respect to time. Again for a more in-depth discussion of Eq. S6, the reader is directed to ref. 5.

2 Clustering Algorithms

2.1 Types of Clusters

Before discussing the differences between some of the most common clustering algorithms, and the specifics of the algorithm employed in this work, it is useful to discuss the different types of clusters that may be identified. An example of different types of clusters in an example two dimensional feature space is given in Fig. S1. In each case, cluster membership is represented by the shared colours of data points, with black points corresponding to data points that cannot be classified and are thus marked as noise. Note, some algorithms may not allow for the identification of outliers, and the definition of noise depends on the algorithm of choice.

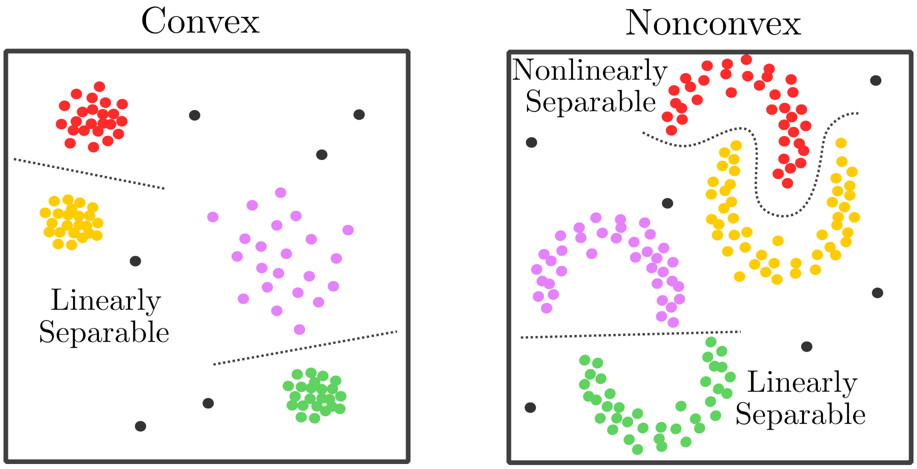

Figure S1 Schematic showing different shapes of clusters in a two-dimensional feature space, with red, yellow, magenta, and green identifying four different clusters and black indicating outlier points. The left panel shows examples of convex clusters that are linearly separable (as indicated by the dashed lines), with both compact (red, yellow, and green) and diffuse (green) clusters. The right panel shows nonconvex clusters, which may not be linearly separable (as is the case for the red and yellow clusters).

The left hand panel represents the simple case of a series of convex clusters that can all be separated by a straight line - i.e. they are linearly separable. Clusters are considered to be convex if all pairs

of points in the cluster can be reached by drawing a straight line between them, without exiting the cluster. Here the group of clusters have different sizes and densities, often it can be difficult to separate clusters in data sets that contain highly variable density. In the right hand panel, we see the more complex case of nonconvex clusters, which may or may not be linearly separable. In the case of the two overlapping ”U” shaped clusters in the upper portion of the panel, these cannot be separated by a linear boundary, and are therefore nonlinearly separable. It is worth noting that not all clustering algorithms are capable of identifying all types of clusters simultaneously. Generally each algorithm is limited in one way or another, for example, one algorithm may be good at identifying clusters that are linearly separable, but not nonlinearly separable. Therefore, the choice in clustering algorithm often depends on the specific type of data set involved.

2.2 Overview of Algorithms

Some of the most widely used clustering algorithms include those based on connectivity, centroid based algorithms, generative probabilistic models, and density based algorithms[6]. Connectivity based approaches, such as agglomerative hierarchical clustering, start by considering all data points as individual clusters. Data points are then merged based on a chosen linkage criterion[7,8], which determines the distance between sets of data points based on a chosen distance metric. A common linkage criterion is single linkage, which merges the set of clusters at each iteration based on the the minimum distance between the two nearest neighbouring data points contained within two separate clusters. This allows clusters to be built up at multiple levels of granularity, however the clustering result may vary dramatically based on the chosen distance metric and linkage criterion. While one can view the clusters at each level of granularity, the optimal number of clusters may not be immediately obvious, and thus require careful inspection of the results at each level. Depending on the chosen linkage criterion, the algorithm may struggle with both nonconvex and nonlinearly separable clusters. Moreover, it can often lead to imbalanced cluster sizes as it prioritises the merging of smaller clusters first. The hierarchical agglomerative algorithms are among the most commonly employed due to their simplicity and general applicability, given a suitable distance

metric is defined.

Centroid based algorithms includes the widely used k-means[9] algorithm. The k-means algorithm proceeds by initialising k centroids randomly, data points are then assigned to the closest centroid and these are updated based on the mean of all points within the respective centroid. This procedure is repeated until the change in location of the centroids does not vary between iterations. While easy to use, k-means is highly sensitive to the initialisation of the centroids, requires the user to specify the number of clusters, and cannot identify nonlinearly separable or nonconvex clusters.

The second category, probabilistic models[10], assume that the data can be modelled by a probability distribution, such as a mixture of Gaussian distributions[11]. The model parameters are refined using the observed data to achieve a maximum likelihood fit, and from this the fit the probabilities of each data point in the model are determined. Such approaches can suffer due to the assumed form of the probability distribution, the need for the refinement of a large number of model parameters, and may struggle with convergence in relation to the algorithms used to estimate these parameters.

Finally, the category of density based clustering algorithms assign clusters based on the local density of data points in the feature space[12]. The definition of density varies, from a number of points in a given local volume to kernel estimates of the density. A major advantage of density based methods is that they can easily detect clusters that are nonconvex and nonlinearly separable, they also often include forms of outlier detection therefore making them fairly robust with respect to noise. For these reasons, we focus on density based algorithms in our application to photoexcited trajectory data. However, it is worth noting at this point that these algorithms can sometimes struggle to identify multiple clusters of highly varying density, which can potentially cause issues in applications to photoexcited trajectories.

2.3 DBSCAN

We shall now take a deeper look at density clustering algorithms, reviewing the basic concepts of the algorithm employed in the current work, DBSCAN[13]. This algorithm involves specifying a

neighbourhood around each individual data point, with points of "core density" including a minimum number of other data points within the radius of this neighbourhood. Clusters are then built up from the regions of core density that overlap and any additional points that border them.

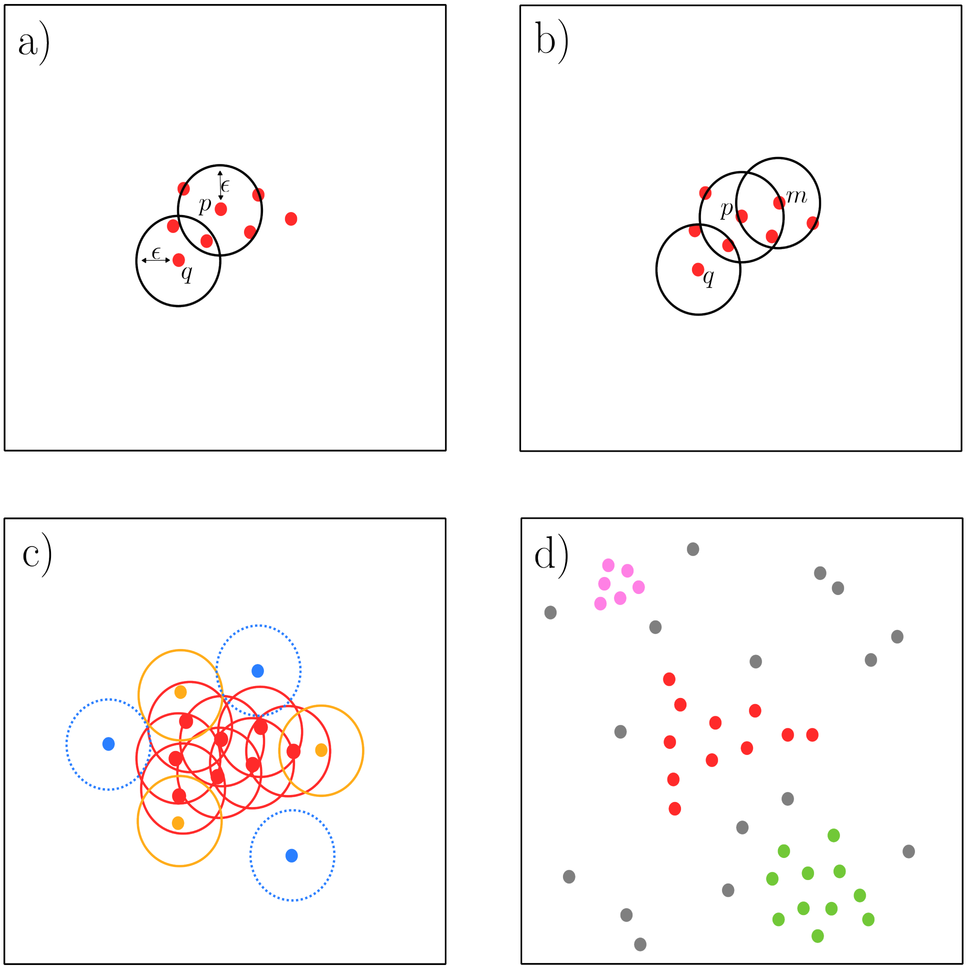

Figure S2 Depiction of the main concepts in DBSCAN. a) two data points p and q with ε defining the area within which other points are directly density reachable. b) two points q and m are density connected by p. c) The building up of core (red), border (yellow) and noise (blue) points. d) Clusters identified by DBSCAN in an example data set. Grey points correspond to noise.

Key to DBSCAN is the idea of density reachability. A data point is said to be directly density reachable from a point p if it is within a radius ε from p, where ε defines the neighbourhood and its size. A point q outside the radius ε, is said to be density reachable from p if there exist a series of successive directly density reachable points between them. Both of these definitions can be seen in Fig. S2a, where all points within ε of p are directly density reachable with respect to p, and q

is density reachable from p as they are connected by a series of directly density reachable points. Furthermore, a point m is said to be density connected to a point q outside of its radius ε if there exists a point p from which both q and m are density reachable, as in Fig. S2b. Using these rules, the algorithm identifies so-called core and border points in the data set. Core points are surrounded by at least $MinPts$ directly density reachable data points within their radius ε. Border points do not require $MinPts$ data points, but must be density connected to a core point. All other points are classified as noise points and marked as outliers. Figure S2c depicts a series of core (red), border (yellow) and noise points (blue) for a simple example. Once all points are built up, clustering is complete as in Fig. S2d. The two parameters ε and $MinPts$ are therefore crucial to the DBSCAN algorithm. By increasing the size of the neighbourhood defined by ε, one can detect more sparsely arranged data at a higher level of granularity. However, this might mean that localised and highly dense clusters may not be detected. Since the size of the density neighbourhood depends on ε, which is defined for all data points, the algorithm is not always capable of detecting clusters of varying densities.

2.4 Identifying Reactive Pathways

In the main text, we demonstrate how algorithms such as DBSCAN can be used to identify key reactive pathways using a pre-computed distance matrix that captures the similarity of trajectories over the simulation period. Each identified cluster corresponds to a unique reactive pathway, or a concentration of reaction flux. In this case, all reactive pathways lead to a unique photoproduct, and so each cluster represents a key path to one possible photoproduct. However, this may not always be the case as certain systems may observe two or more different reactive pathways to the same product. The number of trajectories within each cluster gives some indication of its contribution to the total wavepacket. In simulation methods where the weights of trajectories, *i.e.* the expansion coefficients $c_n(t)$, are all equal in size and time-independent, such as is the case for surface hopping, the percent contribution of each cluster I to the total wavepacket is simply given

by,

$$\%S_I = 100 \times N_I / N_{\text{trj}}, \tag{S7}$$

where N_I is the number of trajectories within the cluster and N_{traj} is the total number of trajectories within the ensemble. In the AIMCE method, the trajectories are initially propagated independently according to Ehrenfest equations of motion and then later coupled a posteriori via the complex and time-dependent expansion coefficients $c_n(t)$ (Eq. S1). In that scenario, a better estimate of the percent importance of each cluster is given by,

$$\%S_I = 100 \times \sum_{n \in I} \frac{1}{T} \int_T c_n(t)\, dt, \tag{S8}$$

where T is the total simulation time and the sum runs over the trajectories contained in cluster I. If a more sophisticated clustering method is used in which trajectories are allowed to enter and leave clusters, this could be accounted for by replacing T by T_{nI}, which is the time that trajectory n is considered to be in cluster I.

3 Dynamic Time Warping

Dynamic time-warping (DTW),[14,15] allows for stretching or contraction of the temporal axis, warping the trajectories non-linearly in time and accounting for slight offsets in their temporal evolution. Furthermore, the potential for a 1:Many mapping of temporal indices allows one to map time series that may observe local changes in frequency, and also compare series of different lengths. A given mapping of temporal indices that describes how the temporal axis is warped to align the two time series, is defined by the warping path. Generally, for a given pair trajectories, there exist a number of possible warping paths. However, we wish to find the warping path that results in optimal alignment of the trajectories such that the alignment cost is minimised.

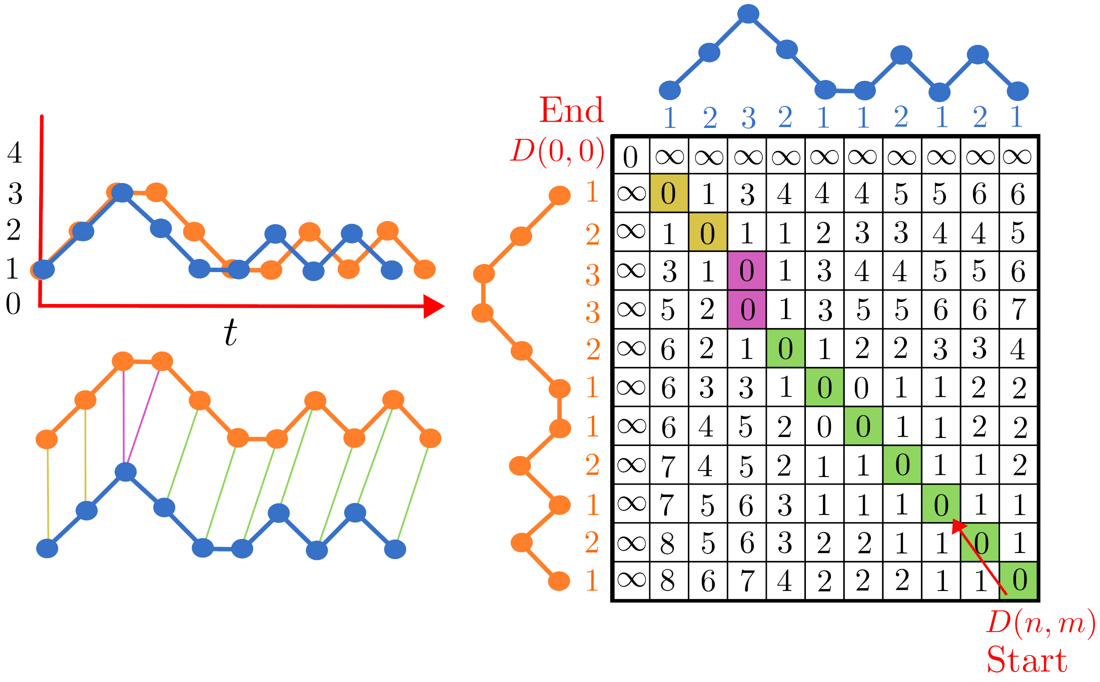

Figure S3 The main concepts of the DTW algorithm. In the top left we see two univariate time series, both are similar but exhibit a small local variation in frequency and are slightly offset in time. On the right hand side we can see the cost matrix D, with elements according to Eq. S9. The elements included in the warping path are coloured according to the type of step taken, and the corresponding alignment can be seen in the bottom left of the figure. Note the magenta vertical steps, which provide a 2:1 mapping of time points. Steps that are coloured green allow the time axis to warp slightly, accounting for small offsets in time.

To demonstrate how the warping path is calculated, let us consider two trajectories o_1 and o_2, with time sets $T_1 = [t_1, t_2, \ldots, t_n]$ and $T_2 = [t_1, t_2, \ldots, t_m]$ respectively. Note, one does not necessarily require $n = m$. Next, we construct a cost matrix D of dimensions $n+1 \times m+1$, in which the calculation of the matrix elements are treated as a dynamic programming problem. The cost matrix contains all possible paths that can be taken to warp the two trajectories in time, with each element comparing two temporal indices. The first row $D(0, 1:m)$ and column $D(1:n, 0)$ are initialised as infinity, and $D(0,0)$ is set to zero. This ensures that the first time step of both time series are always matched together, and is shown for a simple working example on the right hand side of Fig. S3. The elements of the matrix are then calculated as,

$$\begin{aligned} D(i,j) = |o_{1i} - o_{2j}| + \min[&D(i-1, j-1), \\ &D(i-1, j), \\ &D(i, j-1)]. \end{aligned} \tag{S9}$$

with i and j the time indices of each trajectory, running to $i \to n$ and $j \to m$ respectively. Here, the first term is the norm between trajectories o_1 and o_2 at the respective temporal indices. The second term is a referred to as a stepping pattern, corresponding to the minimum value of previously calculated elements. Once the matrix elements have been calculated, the minimum warping path is determined by starting at element $D(n,m)$ and traversing back through the matrix to $D(0,0)$, indicated by the red arrow at element $D(n,m)$ in Fig. S3. The traversal proceeds by selecting the minimum value of elements $D(n-1, m-1)$, $D(n-1, m)$, $D(n, m-1)$ as the next element to be selected in the traversal. Taking the set of three elements in order, these correspond to diagonal, horizontal, and vertical steps through the cost matrix. Taking a diagonal step allows the warping path to bend in time, accounting for temporal distortions such as one trajectory being shifted slightly in time, indicated by both the green and yellow matrix elements in Fig. S3. Here green elements result in a warping of the temporal axis as $i \neq j$, whereas yellow corresponds to the case where the indices matched are at the same point in time i.e. $i = j$. In contrast, horizontal and vertical steps allow one trajectory to skip or repeat a temporal index, respectively. These steps

either speed a trajectory up or slow it down, allowing the potential for a 1:Many mapping of time points. In Fig. S3 vertical steps are indicated in magenta. This provides a way to account for changes in local frequency between a given pair of trajectories. After a step is taken, the direction of the movement is recorded and the procedure is repeated until element $D(0,0)$ is reached. The path taken through the cost matrix defines the warping path, with the sum of all its elements the total cost of alignment. This total cost corresponds to the measure of distance between the two trajectories, with lower values indicating they are more similar. For the example in Fig. S3, the alignment of the two time series defined by the warping path is shown in the lower left hand portion of the figure, with the points coloured according to the type of step taken through the cost matrix.

While the example in Fig. S3 shows the univariate case, DTW can be extended to handle multidimensional time series[15,16]. Generally there are two approaches, the simplest involves independent application of the univariate DTW algorithm to each dimension, the total cost is then the sum of the costs of each independent dimension. Alternatively, one can employ full multidimensional DTW (MD-DTW), which ensures all dimensions warp together by construction of a higher order cost tensor. The warping path is calculated in the same fashion, except now one has to trace through a higher order tensor. As a result, the computational complexity increases due to the associated increase in the number of possible warping paths with the number of dimensions. It has been shown that the case where independent treatment of the dimensions is assumed can lead to wrong classification, and really one should use full MD-DTW where possible[16]. For this reason, we only consider the use of full MD-DTW in this work.

It is worth noting that DTW was originally developed for applications in speech recognition[14]. Since then, DTW has been employed in a wide range of fields, being applied to many different types of time series data[17–22]. In some applications one may not desire frequency invariance or the ability to warp the temporal axis to account for offsets in time. It is possible to tune the degree of this by modifying the stepping pattern given in Eq. S9. For example, one may not consider horizontal steps at all, or limit the number of successive horizontal and vertical steps taken. Moreover, one can bias against certain steps by adding an additional penalty cost to them.

In the context of quantum molecular dynamics, we may wish to limit the flexibility of the warping with respect to frequency, since molecular vibrations of distinct frequency generally correspond to distinct motions. For instance, the same normal mode could have quite different frequencies on different electronic states (potential energy surfaces). In this scenario, fully frequency-invariant DTW could allow quite different motions to be clustered unless the electronic state populations are included as a feature. Nevertheless, DTW allows one to account for the slight offset of two trajectories in time as they follow the same path through phase space, just separated slightly in time. It is thus a question of controlling the degree of DTW, since too much flexibility can render different time scales undetectable and indistinguishable.

Notes and references

(1) Shalashilin, D. V. Quantum mechanics with the basis set guided by Ehrenfest trajectories: Theory and application to spin-boson model. *J. Chem. Phys.* **2009**, *130*, 244101.

(2) Shalashilin, D. V. Nonadiabatic dynamics with the help of multiconfigurational Ehrenfest method: Improved theory and fully quantum 24D simulation of pyrazine. *J. Chem. Phys.* **2010**, *132*, 244111.

(3) Shalashilin, D. V. Multiconfigurational Ehrenfest approach to quantum coherent dynamics in large molecular systems. *J. Comput. Appl. Math.* **2011**, *153*, 105.

(4) Saita, K.; Shalashilin, D. V. On-the-fly ab initio molecular dynamics with multiconfigurational Ehrenfest method. *J. Chem. Phys.* **2012**, *137*, 22A506.

(5) Kirrander, A.; Vacher, M. *Quantum Chemistry and Dynamics of Excited States*; John Wiley & Sons, Ltd, 2020; pp 469–497.

(6) Aggarwal, C. C.; Reddy, C. K. *Data Clustering: Algorithms and Applications*; Chapman and Hall/CRC, 2016.

(7) Nielsen, F. In *Introduction to HPC with MPI for Data Science*; Nielsen, F., Ed.; Undergraduate Topics in Computer Science; Springer International Publishing: Cham, 2016; pp 195–211.

(8) Vinzamuri, C. K. R., Bhanukiran *Data Clustering*; Chapman and Hall/CRC, 2014; Chapter 4, pp 87–110.

(9) Jin, X.; Han, J. In *Encyclopedia of Machine Learning and Data Mining*; Sammut, C., Webb, G. I., Eds.; Springer US: Boston, MA, 2017; pp 695–697.

(10) Jiawei, H.; Deng, H. *Data Clustering*; Chapman and Hall/CRC, 2014; Chapter 3, pp 61–86.

(11) Reynolds, D. In *Encyclopedia of Biometrics*; Li, S. Z., Jain, A., Eds.; Springer US: Boston, MA, 2009; pp 659–663.

(12) Ester, M. *Data Clustering*; Chapman and Hall/CRC, 2014; Chapter 5, pp 111–126.

(13) Ester, M.; Kriegel, H.; Sander, J.; Xu, X. A Density-Based Algorithm for Discovering Clusters in Large Spatial Databases with Noise. Knowledge Discovery and Data Mining. 1996.

(14) Sakoe, H.; Chiba, S. Dynamic programming algorithm optimization for spoken word recognition. *IEEE Trans. Acoust., Speech, Signal Process.* **1978**, *26*, 43–49.

(15) Giorgino, T. Computing and Visualizing Dynamic Time Warping Alignments in R: The dtw Package. *J. Stat. Softw.* **2009**, *31*, 1–24.

(16) Shokoohi-Yekta, M.; Hu, B.; Jin, H.; Wang, J.; Keogh, E. Generalizing DTW to the multi-dimensional case requires an adaptive approach. *Data Min. Knowl. Discov.* **2017**, *31*, 1–31.

(17) Dupas, R.; Tavenard, R.; Fovet, O.; Gilliet, N.; Grimaldi, C.; Gascuel-Odoux, C. Identifying seasonal patterns of phosphorus storm dynamics with dynamic time warping. *Water Resour. Res.* **2015**, *51*, 8868–8882.

(18) Li, K.; Sward, K.; Deng, H.; Morrison, J.; Habre, R.; Franklin, M.; Chiang, Y.-Y.; Ambite, J. L.; Wilson, J. P.; Eckel, S. P. Using dynamic time warping self-organizing maps to characterize diurnal patterns in environmental exposures. *Sci. Rep.* **2021**, *11*, 24052.

(19) Gupta, K.; Chatterjee, N. Financial Time Series Clustering. Information and Communication Technology for Intelligent Systems (ICTIS 2017) - Volume 2. Cham, 2018; pp 146–156.

(20) Aghabozorgi, S.; Seyed Shirkhorshidi, A.; Ying Wah, T. Time-series clustering – A decade review. *Inf. Syst.* **2015**, *53*, 16–38.

(21) Chen, M. Time Series Clustering and Classification. *J. Am. Stat. Assoc.* **2020**, *115*, 1558–1558.

(22) Javed, A.; Lee, B. S.; Rizzo, D. M. A benchmark study on time series clustering. *Machine Learning with Applications* **2020**, *1*, 100001.
